# Aquatic dance flies (Diptera, Empididae, Clinocerinae and Hemerodromiinae) of Greece: species richness, distribution and description of five new species

**DOI:** 10.3897/zookeys.724.21415

**Published:** 2017-12-21

**Authors:** Marija Ivković, Josipa Ćevid, Bogdan Horvat, Bradley J. Sinclair

**Affiliations:** 1 Division of Zoology, Department of Biology, Faculty of Science, University of Zagreb, Rooseveltov trg 6, 10000 Zagreb, Croatia; 2 Zagrebačka 21, 22320 Drniš, Croatia; 3 Canadian National Collection of Insects & Canadian Food Inspection Agency, Ottawa Plant Laboratory – Entomology, Ottawa, Canada; 4 Deceased, formerly with Slovenian Museum of Natural History

**Keywords:** Clinocerinae, Hemerodromiinae, new species, key to species, faunistics, European Ecoregions, Greece

## Abstract

All records of aquatic dance flies (37 species in subfamily Clinocerinae and 10 species in subfamily Hemerodromiinae) from the territory of Greece are summarized, including previously unpublished data and data on five newly described species (*Chelifera horvati* Ivković & Sinclair, **sp. n.**, *Wiedemannia iphigeniae* Ivković & Sinclair, **sp. n.**, *W. ljerkae* Ivković & Sinclair, **sp. n.**, *W. nebulosa* Ivković & Sinclair, **sp. n.** and *W. pseudoberthelemyi* Ivković & Sinclair, **sp. n.**). The new species are described and illustrated, the male terminalia of *Clinocera megalatlantica* (Vaillant) are illustrated and the distributions of all species within Greece are listed. The aquatic Empididae fauna of Greece consists of 47 species, with the following described species reported for the first time: *Chelifera angusta* Collin, *Hemerodromia melangyna* Collin, *Clinocera megalatlantica*, *Kowarzia plectrum* (Mik), *Phaeobalia dimidiata* (Loew), W. (Chamaedipsia) beckeri (Mik), W. (Philolutra) angelieri Vaillant and W. (P.) chvali Joost. A key to species of aquatic Empididae of Greece is provided for the first time. Information related to the European Ecoregions in which species were found is given. Compared to the other studied countries in the Balkans, the Greek species assemblage is most similar to that of the Former Yugoslav Republic of Macedonia.

## Introduction

The aquatic dance flies of the family Empididae (Diptera) comprise the subfamilies Clinocerinae and Hemerodromiinae. Larvae mostly live in aquatic habitats and both larvae and adults are predators, primarily feeding on Simuliidae (Vaillant 1952, 1953, Werner and Pont 2003) and Chironomidae (Vaillant 1967, Harkrider 2000, Ivković et al. 2007). Adult Hemerodromiinae are distinguished by raptorial forelegs and live and hunt in riparian vegetation. On the other hand, adult Clinocerinae are primarily found on the surface of emergent wet stones or in moss mats (Wagner 1997, Ivković et al. 2007).

The aquatic dance fly fauna of Greece has been sporadically investigated during the last few decades. The first records were noted by Vaillant and Wagner (1990), Wagner (1981, 1990, 1995), Wagner and Horvat (1993), and recently by Ivković et al. (2012).

Distribution and diversity studies are of immense importance in studying factors that influence and determine diversity hotspots (Ivković and Plant 2015, Schmidt-Kloiber et al. 2017). The present paper is based on detailed analysis of all publications on Greek aquatic dance flies known to the authors. The authors have also contributed additional records of Greek aquatic dance flies resulting from the examination of specimens collected by colleagues who surveyed 258 sites sampled in the late 1980s and early 1990s. In addition, one new species of *Chelifera* Macquart and four new species of *Wiedemannia* Zetterstedt are herein described.

## Material and methods

**Figure 1.**
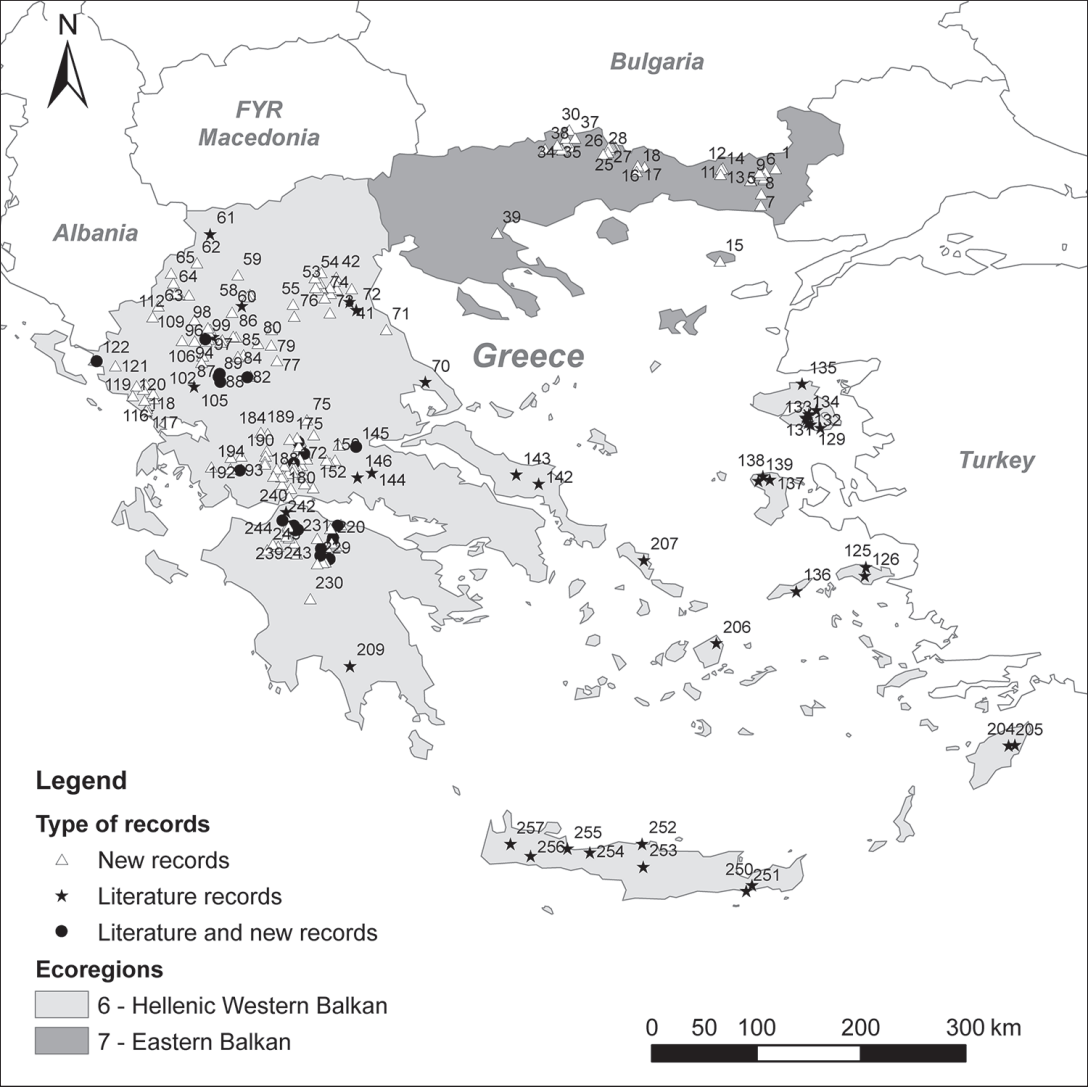
Sampling sites of aquatic Empididae recorded from Greece (see Table 1 for codes).

**Specimen records.** This paper is based on a review of the literature, and primarily on unpublished data and specimens from Bogdan Horvat’s study of the aquatic dance fly fauna of Greece. Wherever possible, each literature record and specimen record was georeferenced as precisely as possible using ArcGIS software. The names of taxa reflect current nomenclature and classifications (Sinclair 1995, Yang et al. 2007). The literature used for identifications included Engel (1939, 1940), Vaillant and Wagner (1990), Wagner (1981, 1990, 1995), Wagner and Horvat (1993) and Ivković et al. (2012). Locality records are listed for each species. A list of locality names including latitude, longitude, altitude and number codes (site ID) for the localities is presented in Table 1 and a map showing the locations of all the georeferenced sites is also provided (Fig. 1). Specimens were collected using sweep nets and by aspirator. They were preserved in 80% ethanol (EtOH). For the purpose of determination, male terminalia were dissected, boiled in 10% KOH and afterwards neutralized with acetic acid, rinsed in water and identified to species level; or they were macerated in hot 85% lactic acid and stored in 80% ethanol along with the remaining body parts in the same tube. In the genitalia illustrations, only the sockets of the setae are shown on the epandrium; the setae are not drawn. All specimens listed in the material examined sections were collected by Bogdan Horvat, Ignac Sivec, Hans Malicky and Reinhard Gerecke. Taxonomic diversity is considered at the level of subfamily, genus, subgenus and species. The European Ecoregions are those of Limnofauna Europaea (Illies 1978), where they are defined at a large European scale and based on the biogeography of aquatic macroinvertebrates.

**Table 1.** List of sampling sites in Greece. European Ecoregions are taken from Illies (1978): Hellenic Western Balkan (6) and Eastern Balkan (7).

Site ID	Site name	Latitude / Longitude	Altitude (m)	Ecoregion
1	Thrace, E of Mega Derio	N41°13'10", E26°03'03"	200	7
2	Thrace, W of Mega Derio	N41°11'29", E25°57'30"	710	7
3	Thrace, Lesitse Mts.	N41°07'28", E25°57'04"	760	7
4	Thrace, E of Sapka Mts., big stream in the valley	N41°08', E25°57'	600	7
5	Thrace, N of Avas	N41°00'07", E25°55'36"	200	7
6	Thrace, Sapka Mts. 1	N41°09'56", E25°55'17"	735	7
7	Thrace, 3 km N of Alexandroupoli	N40°54', E25°55'	100	7
8	Thrace, Sapka Mts. 2	N41°11'02", E25°54'40"	545	7
9	Thrace, Sapka Mts., Nea Sanda 1	N41°07'02", E25°50'02"	200	7
10	Thrace, Sapka Mts., Nea Sanda 2	N41°07'06", E25°49'43"	220	7
11	Thrace, Anatoliki Rodopi, E od Drimi	N41°13'26", E25°35'35"	240	7
12	Thrace, Anatoliki Rodopi, Drimi	N41°12'52", E25°34'34"	180	7
13	Thrace, Anatoliki Rodopi, E of Gratini 1	N41°10'10", E25°34'29"	100	7
14	Thrace, Anatoliki Rodopi, E of Gratini 2	N41°10'10", E25°34'29"	160	7
15	Thrace, Samothrace, hygropetric at the church of Kreminotissa	N40°25', E25°34'	400	7
16	Thrace, Miki	N41°14', E24°55'	340	7
17	Thrace, 8 km N of Sminthi	N41°14'49", E24°51'44"	300	7
18	Thrace, N of Xanthi	N41°11'39", E24°51'08"	200	7
19	Thrace, N of Dipotama 1	N41°24'28", E24°40'10"	1430	7
20	Thrace, N of Dipotama 2	N41°24'50", E24°38'51"	1310	7
21	Thrace, Dit. Rodopi, N of Dipotama 1	N41°25'07", E24°38'22"	1290	7
22	Thrace, N of Dipotama 3	N41°23'53", E24°38'06"	1030	7
23	Thrace, N of Dipotama 4	N41°24'47", E24°37'56"	1340	7
24	Thrace, N of Dipotama 5	N41°24'24", E24°37'19"	1400	7
25	Thrace, Dit. Rodopi, N of Dipotama 2	N41°23', E24°37'	1000	7
26	Thrace, Dit. Rodopi, N of Dipotama 3	N41°24'14", E24°36'45"	1415	7
27	Thrace, S of Dipotama	N41°21'22", E24°36'20"	440	7
28	Thrace, S of Silli	N41°20'40", E24°33'50"	315	7
29	Thrace, Rodopi, Skaloti	N41°24', E24°17'	1090	7
30	Thrace, Rodopi 1	N41°33'00", E24°16'25"	1400	7
31	Thrace, N of Sidironero 1	N41°26'42", E24°14'46"	930	7
32	Thrace, Rodopi 2	N41°28'48", E24°14'40"	945	7
33	Thrace, N of Sidironero 2	N41°22'50", E24°13'04"	910	7
34	Thrace, W of Sidironero	N41°23'13", E24°12'03"	500	7
35	Thrace, Rodopi, E of Mikromilia	N41°25'22", E24°10'04"		670	7
36	Thrace	Unspecified		7
37	Macedonia, Dit. Rodopi, Elatia forest	N41°29', E24°19'	1450	7
38	Macedonia, E of Mikroklisoura	N41°23'14", E24°03'48"	370	7
39	Macedonia, N of Stavros	N40°40', E23°39'	100	7
40	Macedonia, R. Mavroneri, 10 km W of Katerini	N40°11', E22°24'	160	6
41	Macedonia, Olympus Mts. above Agios Dyonysos, Prionia	N40°04', E22°22'	1050–1700	6
42	Macedonia, Pieria Mts., 2 streams on Ritini	N40°17', E22°16'	800	6
43	Macedonia, N of Agios Dimitrios	N40°10', E22°16'	660	6
44	Macedonia, Pieria Mts., S of Elatohori	N40°15', E22°15'	1010	6
45	Macedonia, S of Agios Dimitrios	N40°08'37", E22°13'07"	860	6
46	Macedonia, Pieria Mts., E of Fteri	N40°11'52", E22°12'42"	840	6
47	Macedonia, Pieria Mts., Fteri	N40°11', E22°09'	1080	6
48	Macedonia, Pieria Mts., W of Fteri	N40°11'49", E22°08'20"	1440	6
49	Macedonia, W of Daskio	N40°19'32", E22°08'14"	460	6
50	Macedonia, Pieria Mts., E of Velventos	N40°14'05", E22°07'51"	1330	6
51	Macedonia, Pieria Mts., 1	N40°10'35", E22°06'54"	1500	6
52	Macedonia, Pieria Mts., 2	N40°13'42", E22°06'37"	1270	6
53	Macedonia, Pieria Mts., 3	N40°11'35", E22°05'31"	1480	6
54	Macedonia, E of Velventos	N40°16'54", E22°05'11"	420	6
55	Macedonia, Phalacro Mts., N of Livadero	N40°03', E21°53'	690	6
56	Macedonia, Grevena, Milea	N40°08', E21°31'	480	6
57	Macedonia, Grevena, 6 km S of Milea	N40°07', E21°30'	470	6
58	Macedonia, Grevena, stream S of R. Aliakmon by Kamilas Pigi	N40°02', E21°27'	600	6
59	Macedonia, Kozani, Polilako (Paraveti), Neapolis	N40°18', E21°25'	550	6
60	Macedonia, Grevena, R. Venetikos, Kipourio	N39°59', E21°22'	500	6
61	Macedonia, Vernon, influx of Aliakmon between Gavros and Aposkepos	N40°39', E21°11'	450	6
62	Macedonia, Kastoria, Nestorio	N40°24', E21°04'	800	6
63	Macedonia, Smokilas Mts., main stream near the bridge, 2 km E of Agia Paraskevi	N40°08', E21°00'	1100	6
64	Macedonia, Kastoria, Grammos Mts., 7 km S Chrisi	N40°14', E20°52'	650	6
65	Macedonia, Kastoria, Grammos Mts., 6 km N Pefkofito	N40°19', E20°50'	1500	6
66	Macedonia, Chalkidiki, Chlomon Oros., Paleokastron, Vatonia P. 1	Unspecified		550	/
67	Macedonia, Chalkidiki, Chlomon Oros., Paleokastron, Vatonia P. 2	Unspecified		1500	/
68	Macedonia, Chalkidiki, Chlomon Oros., valley on the southern slope	Unspecified		650	/
69	Macedonia, Xanthi, NE Pass Str. Xanthi-Stavroupolis	Unspecified		800	7
70	Thessaly, Portaria	N39°23', E23°01'	700	6
71	Thessaly, Ossa Mts., stream Apataniana	N39°50', E22°42'	1200	6
72	Thessaly, Karya	N40°00', E22°26'	750–800	6
73	Thessaly, S of Kallithea	N39°58'35", E22°12'49"	510	6
74	Thessaly, Pieria Mts., S of Livadi	N40°06'20", E22°10'11"	800	6
75	Thessaly, 5 km W of Palea Giannitsou	N39°03', E22°01'	500	6
76	Thessaly, Deskati	N39°56'53", E21°54'30"	690	6
77	Thessaly, Trikala, Longiai	N39°34', E21°45'	100	6
78	Thessaly, S of Asprokklisia	N39°49'56", E21°42'48"	500	6
79	Thessaly, Trikala, Moshofito, Avra	N39°42', E21°42'	200	6
80	Thessaly, Kalambaka, Agios Nikolaos	N39°43', E21°35'	200	6
81	Thessaly, Trikala, Stournareika	N39°26', E21°31'	400	6
82	Thessaly, Trikala, Kato Palagokaria	N39°25', E21°30'	600	6
83	Thessaly, Kalambaka, 5 km E of Paleochori	N39°37', E21°28'	600	6
84	Thessaly, Kalambaka, Paleochori	N39°36', E21°25'	1000	6
85	Thessaly, Kalambaka, Trigona	N39°46', E21°24'	400	6
86	Thessaly, Kalambaka, Koridallos	N39°46', E21°22'	450	6
87	Thessaly, Trikala, Arta, Pahtouri	N39°27', E21°16'	600	6
88	Thessaly, Trikala, Arta, R. Ahelos, Kapsala	N39°22', E21°16'	500	6
89	Thessaly, Trikala, Arta, Korifi	N39°25', E21°15'	600	6
90	Thessaly, Trikala, 9 km S of Chrisomilea	Unspecified		6
91	Thessaly, Kalambaka, 4 km S of Ambelia	Unspecified		6
92	Epirus, Metsovo, 14 km S of Milea	N39°44', E21°17'	900	6
93	Epirus, Metsovo, Lakmos Mts., Anilio (5 km S bellow river)	N39°43', E21°16'	1300	6
94	Epirus, Pindus Mts., Metsovo, meadow source easthang	N39°46', E21°12'	1350	6
95	Epirus, N of Katarapass, 1 km SW Milea	N39°50', E21°11'	1300	6
96	Epirus, Metsovo, Katara Pass	N39°48', E21°10'	1350	6
97	Epirus, Metsovo, Lakmos Mts., 2 km S of Anilio (bellow left tributary)	N39°44', E21°10'	840	6
98	Epirus, Metsovo, 12 km W Milea	N39°51', E21°09'	1250	6
99	Epirus, Metsovo, R. Metsovitikos	N39°44', E21°09'	800	6
100	Epirus, Metsovo, Lakmos Mts., Anthohori, (bellow rapid river)	N39°44', E21°08'	780	6
101	Epirus, Lakmos Mts., 10 km S of Anilio	N39°36', E21°07'	1150	6
102	Epirus, Metsovo, Lakmos Mts., Anilio (15 km S influx)	N39°33', E21°06'	500	6
103	Epirus, Metsovo, 14 km W of Milea	N39°55', E21°03'	1000	6
104	Epirus, Ioannina, Megalo Peristeri	N39°44', E21°03'	600	6
105	Epirus, Xerovouni Mts., Plaka, R. Arachthos, u. Agnatha	N39°20', E21°02'	200	6
106	Epirus, Ioannina, R. Zagoritikos, Karies	N39°44', E20°56'	500	6
107	Epirus, Konitsa, Smolikas Mts., Pournia	N40°08', E20°54'	1100	6
108	Epirus, Konitsa, R. Saradaporos, Drosopigi	N40°08', E20°53'	900	6
109	Epirus, Konitsa, Asimohori	N40°02', E20°44'	450	6
110	Epirus, 10 km N of Louros	N39°14'22", E20°42'05"	200	6
111	Epirus, S of Seriziana	N39°17'07", E20°41'37"	200	6
112	Epirus, Ioannina, R. Voidomatis, Aristi	N39°56', E20°41'	400	6
113	Epirus, Preveza, Zalongu, stream 2 km E of Mirsini	N39°07', E20°39'	180	6
114	Epirus, W of Kriopigi	N39°09'30", E20°38'18"	170	6
115	Epirus, R. Aheron, N of Gliki	N39°21'34", E20°37'52"	200	6
116	Epirus, Kanallaki, Skepaston	N39°13', E20°37'	100	6
117	Epirus, Mirsini	N39°08', E20°37'	120	6
118	Epirus, R. Aheron, Gliki	N39°19', E20°36'	50	6
119	Epirus, R. Kokitos, W of Gardiki	N39°21', E20°33'	50	6
120	Epirus, R. Kokitos, Themelo	N39°15', E20°31'	40	6
121	Epirus, Igoumenitsa, Thesprotia, R. Thiamis, Neohori	N39°31', E20°22'	30	6
122	Epirus, Igoumenitsa, R. Thiamis, Soulopoulo	N39°33', E20°12'	5	6
123	Epirus, Ioannina, R. Vardas, Abelos	Unspecified		6
124	Epirus, Ioannina, Balndouma	Unspecified		6
125	North Aegean islands, Samos, below Manolates	N37°47', E26°49'	160	6
126	North Aegean islands, Samos, E of Pirgos	N37°43', E26°49'	300	6
127	North Aegean islands, Lesbos, 7 km E of Plomari	N38°59', E26°26'	110	6
128	North Aegean islands, Lesbos, 1 km W of Ippion	N39°08', E26°24'	70	6
129	North Aegean islands, Lesbos, 1 km SW of Megalochori	N39°01', E26°21'	280	6
130	North Aegean islands, Lesbos, 3 km NW of Agiasos	N39°06', E26°20'	320	6
131	North Aegean islands, Lesbos, 4 km W of Agiasos	N39°06', E26°20'	400	6
132	North Aegean islands, Lesbos, 2 km N of Akrassi	N39°03', E26°19'	370	6
133	North Aegean islands, Lesbos, S of Neochorion	N39°01', E26°19'	270	6
134	North Aegean islands, Lesbos, Ambeliko	N39°04', E26°18'	340	6
135	North Aegean islands, Lesbos, E of Lepetimnos	N39°22', E26°16'	330	6
136	North Aegean islands, Icaria, W of Chrisostomos	N37°35', E26°13'	270	6
137	North Aegean islands, Chios, 2 km N of Fita	N38°32', E26°00'	510	6
138	North Aegean islands, Chios, N of Keramos	N38°34', E25°56'	60	6
139	North Aegean islands, Chios, 5 km N of Pirama	N38°32', E25°54'	170	6
140	North Aegean islands, Icaria	Unspecified		6
141	North Aegean islands, Lesbos	Unspecified		6
142	Central Greece, Euboea, S of Komiton	N38°30', E24°00'	540	6
143	Central Greece, Euboea, Steni Dirfyos (former Ano Steni)	N38°35', E23°49'	550	6
144	Central Greece, Polydrosos	N38°36', E22°34'	1060–1250	6
145	Central Greece, Etolia, Lamia, Ieraklia	N38°49', E22°26'	25	6
146	Central Greece, Parnassus Mts., above Polydrosos	N38°33', E22°26'	1000	6
147	Central Greece, Oeta Mts., between Kastanea and Katafygio	N38°50', E22°17'	1400	6
148	Central Greece, Etolia, Vardousia Mts., Stromi	N38°42', E22°15'	820	6
149	Central Greece, Etolia, Vardousia Mts., Mousonitsa	N38°41', E22°12'	650	6
150	Central Greece, Etolia, Vardousia Mts., Athanasios Diakos	N38°42', E22°11'	830	6
151	Central Greece, Etolia, Vardousia Mts., Paleovraha	N38°55', E22°04'	170	6
152	Central Greece, Etolia, Nafpaktos, 9 km S of Krokilio	N38°28', E22°04'	1000	6
153	Central Greece, Etolia, Vardousia Mts., 5 km N of Grammeni Oxia	N38°45', E22°00'	1150	6
154	Central Greece, Etolia, Vardousia Mts., R. Evinos, Grammeni Oxia	N38°43', E22°00'	800	6
155	Central Greece, Etolia, Vardousia Mts., 9 km N of Grammeni Oxia	N38°47', E21°59'	1050	6
156	Central Greece, Etolia, Vardousia Mts., 7 km N of Grammeni Oxia	N38°46', E21°59'	1400	6
157	Central Greece, Etolia, Vardousia Mts., 7 km S of Gardiki	N38°45', E21°59'	1300	6
158	Central Greece, Etolia, Vardousia Mts., Terpsithea	N38°33', E21°59'	570	6
159	Central Greece, Etolia, Nafpaktos, R. Mornos, Limnitsa	N38°30', E21°59'	200	6
160	Central Greece, Etolia, Vardousia Mts., Elatovrisi	N38°39', E21°58'	750	6
161	Central Greece, Etolia, Vardousia Mts., Elato	N38°35', E21°58'	1000	6
162	Central Greece, Etolia, Vardousia Mts., 6 km S of Lefkada	N38°52', E21°57'	500	6
163	Central Greece, Etolia, Vardousia Mts., Gardiki	N38°51', E21°57'	580	6
164	Central Greece, Etolia, Vardousia Mts., 13 km S of Gardiki	N38°43', E21°57'	700	6
165	Central Greece, Etolia, Vardousia Mts., Pougkakia	N38°51', E21°56'	600	6
166	Central Greece, Etolia, Vardousia Mts., 2 km W of Gardiki	N38°49', E21°56'	1100	6
167	Central Greece, Etolia, Vardousia Mts., Grigorio	N38°38', E21°56'	1400	6
168	Central Greece, Tymfristos Mts., R. Sperhios, Lamia	N38°54', E21°55'	550	6
169	Central Greece, Etolia, Vardousia Mts., Ano Chora	N38°36', E21°55'	700	6
170	Central Greece, Etolia, Panaitoliko Mts., Klepa	N38°41', E21°54'	700	6
171	Central Greece, Etolia, Panaitoliko Mts., R. Evinos, Klepa	N38°40', E21°54'	500	6
172	Central Greece, Etolia, Vardousia Mts., 3 km W of Kryoneri	N38°38', E21°54'	1100	6
173	Central Greece, Etolia, Vardousia Mts., Kato Chora	N38°36', E21°53'	600	6
174	Central Greece, Etolia, Nafpaktos, Anthofito	N38°28', E21°52'	100	6
175	Central Greece, Karpenisi, Agios Nikolaos	N38°53', E21°51'	1000	6
176	Central Greece, Etolia, Nafpaktos, tributory of R. Evinos, 6 km N of Pokista	N38°35', E21°51'	460	6
177	Central Greece, Etolia, R. Mornos, Nafpaktos	N38°23', E21°51'	10	6
178	Central Greece, Etolia, Agrinio, Panaitoliko Mts., R. Evinos, Agios Dimitros	N38°39', E21°49'	400	6
179	Central Greece, Etolia, Nafpaktos, 2 km N of Pokista	N38°34', E21°48'	350	6
180	Central Greece, Etolia, Nafpaktos, Simos	N38°30', E21°48'	350	6
181	Central Greece, Etolia, Nafpaktos, Pokista	N38°34', E21°47'	370	6
182	Central Greece, Etolia, Agrinio, Peristra, 1 km S of Perkos	N38°38', E21°45'	300	6
183	Central Greece, Etolia, Agrinio, R. Evinos, Kato Hrisovitsa, Diasellaki	N38°34', E21°43'	230	6
184	Central Greece, Panaitoliko Mts., R. Tavropos, Kalesmeno	N38°56', E21°40'	300	6
185	Central Greece, Etolia, Agrinio, Panaitoliko Mts. R. Trikeriotis, Dermatio	N38°47', E21°40'	400	6
186	Central Greece, Etolia, Panaitoliko Mts., Prousos	N38°44', E21°39'	660	6
187	Central Greece, Etolia, Panaitoliko Mts., Chaliki, Ladikon	N38°41', E21°39'	900	6
188	Central Greece, Etolia, Panaitoliko Mts., Chaliki, Nerosirtis	N38°40', E21°39'	750	6
189	Central Greece, Etolia, Agrinio, Panaitoliko Mts., Anatoliki Frangista	N38°56', E21°37'	800	6
190	Central Greece, Etolia, Agrinio, Panaitoliko Mts., Potamoula	N38°44', E21°26'	200	6
191	Central Greece, Etolia, Agrinio, Agia Soufia	N38°36', E21°26'	100	6
192	Central Greece, Etolia, Lamia, Pavliani	N38°44', E21°21'	100	6
193	Central Greece, Etolia, Agrinio, Panaitoliko Mts., Megali Chora	N38°38', E21°21'	40	6
194	Central Greece, Etolia, Giona Mts., Sikia	N38°38', E21°11'	510	6
195	Central Greece, Oeta Mts., stream Valorema, Pavliani	Unspecified		1600	6
196	Central Greece, Etolia, Agrinio, Ahlavokastro	Unspecified		6
197	Central Greece, Etolia, Arta, Loutraki	Unspecified		6
198	Central Greece, Etolia, Agrinio, Panaitoliko Mts., Houni	Unspecified		6
199	Central Greece, Etolia, Agrinio, Panaitoliko Mts., Palagohori	Unspecified		6
200	Central Greece, Etolia, Nafpaktos, Avrorema bridge	Unspecified		6
201	Central Greece, Central Euboea	Unspecified		6
202	Central Greece, Etolia, Agrinio, Panaitoliko Mts., 3 km N of Hani Lioliou	Unspecified		6
203	Central Greece, Etolia, Nafpaktos, Koutsopanneika	Unspecified		6
204	Dodecanese islands, Rhodes, 3 km E of Archipolis	N36°15', E28°06'	100	6
205	Dodecanese islands, Rhodes, near Archipolis	N36°15', E28°03'	200	6
206	Cyclades islands, Naxos, S of Koronis	N37°08', E25°32'	630	6
207	Cyclades islands, Andros, Apikia	N37°51', E24°54'	220	6
208	Cyclades islands, Andros	Unspecified		6
209	Peloponnese, Taygetos Mts. (below summit)	N36°56', E22°23'	900	6
210	Peloponnese, village Akrata	N38°09', E22°18'	80	6
211	Peloponnese, R. Krathis, Voutsimos	N38°08', E22°16'	160	6
212	Peloponnese, Aroania Mts., 2 km S of Zarouchla	N37°58', E22°16'	1200	6
213	Peloponnese, 3 km N of Agia Varvara	N38°01', E22°15'	900	6
214	Peloponnese, R. Krathis, 7 km N of Peristera	N38°05', E22°14'	600	6
215	Peloponnese, tributary of R. Krathis, 7 km N of Peristera	N38°03', E22°14'	720	6
216	Peloponnese, 2 km N of Peristera	N38°02', E22°14'	800	6
217	Peloponnese, R. Krathis, Peristera	N38°00', E22°14'	1000	6
218	Peloponnese, Aroania Mts., 4 km S of Solos	N37°59', E22°14'	1250	6
219	Peloponnese, Ano Potames, Kalivitis	N38°07', E22°13'	670	6
220	Peloponnese, Aroania Mts., Zarouhla	N37°59', E22°13'	1100	6
221	Peloponnese, Aroania Mts., below Xelmos , Valtos, Zarelia	N37°55', E22°12'	830	6
222	Peloponnese, Likouria (below village)	N37°51', E22°12'	700	6
223	Peloponnese, Aroania Mts., Kalivia	N37°50', E22°10'	470	6
224	Peloponnese, Aroania Mts., Krinofita	N37°49', E22°10'	460	6
225	Peloponnese, Pagrati	N37°49', E22°09'	450	6
226	Peloponnese, Aroania Mts., Kastria	N37°56', E22°08'	670	6
227	Peloponnese, Kato Klitoria	N37°53', E22°08'	500	6
228	Peloponnese, Aroania Mts., Xelmos (above)	N38°02', E22°06'	700	6
229	Peloponnese, Labia Mts., Amigdalia	N37°49', E22°06'	440	6
230	Peloponnese, R. Piro, Elliniko	N37°30', E22°02'	220	6
231	Peloponnese, Panachaiko Mts., tributory of R. Selinous, Leontio	N38°06', E21°56'	700	6
232	Peloponnese, Panachaiko Mts., Leontio	N38°06', E21°55'	640	6
233	Peloponnese, Erymanthos Mts., Lechouri	N37°54', E21°55'	660	6
234	Peloponnese, Panachaiko Mts., Veteika	N38°08', E21°54'	970	6
235	Peloponnese, Erymanthos Mts., Kato Vlasia	N38°00', E21°54'	740	6
236	Peloponnese, Panachaiko Mts., Kounaveika (near village)	N38°08', E21°53'	950	6
237	Peloponnese, Panachaiko Mts., Moira	N38°09', E21°51'	750	6
238	Peloponnese, Panachaiko Mts., Moira (after village)	N38°08', E21°51'	800	6
239	Peloponnese, Erymanthos Mts., Profitis Ilias	N38°02', E21°51'	480	6
240	Peloponnese, Ano Kastritsi, stream	N38°16', E21°50'	500	6
241	Peloponnese, Erymanthos Mts., Stavrohori, Eliniko	N38°03', E21°50'	380	6
242	Peloponnese, Panachaiko Mts., Souli	N38°11', E21°48'	380	6
243	Peloponnese, Erymanthos Mts., S of Spartia	N37°58', E21°46'	800	6
244	Peloponnese, Erymanthos Mts., Manesi	N37°59', E21°43'	350	6
245	Peloponnese, Stavrodromi	N37°56', E21°40'	280	6
246	Peloponnese, Abelokipi	Unspecified		6
247	Peloponnese, E of Olympia	Unspecified		630	6
248	Peloponnese, Panachaiko Mts., Kristalovrisi (stream)	Unspecified		6
249	Peloponnese, Erymanthos Mts., Kalamata	Unspecified		6
250	Crete, E of Agios Ioannis	N35°03', E25°50'	400	6
251	Crete, E of Ierepetra	N35°00', E25°47'	0	6
252	Crete, stream next to Sises	N35°24', E24°54'	50	6
253	Crete, Passas valley near Pass	N35°12', E24°54'	1300	6
254	Crete, S of Retimnon	N35°20', E24°27'	230	6
255	Crete, Georgioupolis	N35°22', E24°15'	0	6
256	Crete, Xyloskalon	N35°18', E23°56'	620	6
257	Crete, stream near Kotsifiana	N35°24', E23°45'	500	6
258	Laschtabend (Alpen)	Unspecified		1200	/

Label data for primary types are cited from the top of the pin downward, with the data from each label in quotation marks. Labels are cited in full, with original spelling, punctuation, and dates, and label lines are delimited by a slash (/). Additional information is included in square [] brackets. The repository of each type is given in parentheses. Secondary type data are abridged and listed alphabetically. This study is based on material housed in the following institutions: Canadian National Collection of Insects, Ottawa, Canada (CNC); col. M. Ivković, University of Zagreb, Croatia (UZC); col. Empididae, Slovenian Museum of Natural History, Ljubljana, Slovenia (SMNH). Terms used for adult structures primarily follow those of Cumming and Wood (2009), except for the antenna and wing venation, where the terminologies of Stuckenberg (1999) and Saigusa (2006) are used, respectively. In the system outlined by Saigusa (2006), the dipteran wing vein A_1_ (as used in McAlpine 1981) is homologized with the mecopteran vein CuP, and consequently CuA_1_ (of McAlpine) is termed M_4_, whereas CuA_2_ is CuA, the anal cell is cell cua and the anal vein (A_1_+CuA_2_) is CuA+CuP. Homologies of the male terminalia follow those of Sinclair and Cumming (2006). Species of *Wiedemannia* described herein will not be assigned to a subgenus because we consider current subgeneric concepts confused and mostly not monophyletic (Ivković et al. 2012).

**Data analysis.** A list of species was compiled from all specimen data (Table 2). Comparison of species richness and assemblage composition with published records from studied countries in the Balkans (Slovenia, Croatia, Bosnia & Herzegovina, Montenegro and Former Yugoslav Republic of Macedonia) was conducted by compiling species lists for those countries taken from Wagner (1981, 1995), Horvat (1993, 1995a, 1995b, 1997) and Ivković et al. (2013a, 2013b, 2014). A species by country matrix was constructed and the Sørensen Index of Similarity of each pairwise comparison (Table 3) was calculated using the Primer v6 software (Clarke and Gorley 2006).

**Table 2.** List of Greek aquatic dance flies and summary of their distribution. European Ecoregions are taken from Illies (1978): Hellenic Western Balkan (6) and Eastern Balkan (7).

Species	Distribution	Ecoregion
Hemerodromiinae		
*Chelifera angusta* Collin, 1927	Europe, Asia	6
*Chelifera barbarica* Vaillant, 1982	Southern Europe, North Africa	6
*Chelifera horvati* sp. n.	Greece	6
*Chelifera precabunda* Collin, 1961	Widespread in Europe	6, 7
*Chelifera precatoria* (Fallén, 1816)	Widespread in Europe	6
*Chelifera stigmatica* (Schiner, 1862)	Widespread in Europe	6, 7
*Chelifera trapezina* (Zetterstedt, 1838)	Widespread in Europe	6
*Hemerodromia melangyna* Collin, 1927	Europe	6
*Hemerodromia oratoria* (Fallén, 1816)	Widespread in Europe, Asia	6, 7
*Hemerodromia unilineata* Zetterstedt, 1842	Europe	6, 7
Clinocerinae		
*Clinocera megalatlantica* (Vaillant, 1957)	Greece, Morocco	7
*Clinocera nigra* Meigen, 1804	Europe, North Africa, Asia	6
*Clinocera stagnalis* (Haliday, 1833)	Europe, North Africa, Asia, and northern North America	6, 7
*Clinocerella siveci* (Wagner & Horvat, 1993)	Greece	6
*Dolichocephala cretica* Wagner, 1995	Greece (Crete)	6
*Dolichocephala guttata* (Haliday, 1833)	Widespread in Europe	6, 7
*Dolichocephala ocellata* (Costa, 1854)	Europe, North Africa	6
*Dolichocephala vaillanti* Wagner, 1995	Greece (Crete)	6
*Dolichocephala zwicki* Wagner, 1995	Balkan region, Greece Islands	6
*Kowarzia barbatula* (Mik, 1880)	Europe, Asia Minor	6, 7
*Kowarzia bipunctata* (Haliday, 1833)	Widespread in Europe, North Africa	6, 7
*Kowarzia madicola* (Vaillant, 1965)	Central and southern Europe	6
*Kowarzia plectrum* (Mik, 1880)	Europe, Asia Minor	6
*Phaeobalia dimidiata* (Loew, 1869)	Europe	6, 7
*Roederiodes malickyi* Wagner, 1981	Greece (Crete)	6
Wiedemannia (Chamaedipsia) aequilobata Mandaron, 1964	Southern Europe	6
Wiedemannia (Chamaedipsia) ariadne Wagner, 1981	Balkan region, Greece Islands	6
Wiedemannia (Chamaedipsia) beckeri (Mik, 1889)	Europe	7
Wiedemannia (Chamaedipsia) lota Walker, 1851	Europe, Asia	6, 7
Wiedemannia (Eucelidia) zetterstedti (Fallén, 1826)	Europe, Asia Minor	6, 7
Wiedemannia (Philolutra) angelieri Vaillant, 1967	Southern Europe	6
Wiedemannia (Philolutra) chvali Joost, 1981	Russia (Kabardino-Balkaria), Greece	7
Wiedemannia (Philolutra) fallaciosa (Loew, 1873)	Europe, Asia Minor, Middle East, North Africa	6, 7
Wiedemannia (Pseudowiedemannia) lamellata (Loew, 1869)	Europe	6, 7
Wiedemannia (Pseudowiedemannia) microstigma (Bezzi, 1904)	Balkan region	6
Wiedemannia (Roederella) czernyi (Bezzi, 1905)	Southern Europe	7
Wiedemannia (Wiedemannia) andreevi Joost, 1982	Balkan region, Poland	6
Wiedemannia (Wiedemannia) bilobata Oldenberg, 1910	Central and southern Europe	6
Wiedemannia (Wiedemannia) dinarica Engel, 1940	Balkan region	6
Wiedemannia (Wiedemannia) dyonysica Wagner, 1990	FYR Macedonia, Greece	6
Wiedemannia (Wiedemannia) graeca Vaillant & Wagner, 1990	Greece	6, 7
Wiedemannia (Wiedemannia) tricuspidata (Bezzi, 1905)	Central and southern Europe	6, 7
*Wiedemannia artemisa* Ivković & Plant, 2012	Balkan region	6
*Wiedemannia iphigeniae* sp. n.	Greece	6
*Wiedemannia ljerkae* sp. n.	Greece	6
*Wiedemannia nebulosa* sp. n.	Greece	7
*Wiedemannia pseudoberthelemyi* sp. n.	Greece	6

## Taxonomy

### Clinocerinae

#### Wiedemannia iphigeniae

Taxon classificationAnimaliaDipteraEmpididae

Ivković & Sinclair sp. n.

http://zoobank.org/584FDF48-D85B-4953-9F7E-079DF489B9C5

Figs 1 , 6 , 7

##### Type locality.

Greece: Peloponnese, Aroania Mts., Krinofita, 37°49'00"N, 22°10'00"E.

##### Type material.

**Holotype** ♂, labelled: “GREECE, Peloponnese/ Aroania Mts., Krinofita/ 37°49'00"N, 22°10'00"E/ 20.iv.1990/ leg. B. Horvat, I. Sivec”; “HOLOTYPE/ *Wiedemannia*/ *iphigeniae*/ Ivković & Sinclair” (CNC, dried from alcohol).

##### Diagnosis.

This species of *Wiedemannia* is distinguished by the apically pointed unilobed cercus with small basal projection and a narrow pterostigma on the wings.

##### Description.

**Male.** Body length approx. 3.5 mm (holotype dissected prior to measurement), wing length 3.7 mm (colouration bleached by prolonged storage in alcohol). Head in lateral view higher than long; gena narrow, nearly one-third height of eye. Frons short, broader than face. Face wide, with distinct carina on lower margin, bare, lacking setae. One pair of ocellar and one pair of vertical setae; about 5 distinct upper postoculars, subequal in size; lower postocular setae finer and merging with longer setae on middle and lower occiput; many setulae present on vertex and between ocellar area. Antenna brownish; postpedicel and stylus minutely pubescent; pedicel slightly longer than scape; scape with complete circlet of subapical setae; postpedicel apically pointed; stylus nearly twice length of postpedicel; scape with setulae dorsally.

Scutum with pale central vitta between dorsocentral rows. Mesonotum with 5 dorsocentral setae, with short setulae intermixed. Acrostichal setae small and fine, biserial, extending to 2^nd^ dorsocentral seta; 1 strong postpronotal seta and 1–4 short setulae; 2 notopleural setae and several setulae; 1 presutural supra-alar seta and many small anterior setulae; 1 postalar seta. Antepronotum with 1 pair of strong setae and 1 pair of smaller setae. Proepisternum with some fine setulae. Laterotergite with several fine, pale setulae. One pair of strong marginal scutellar setae; disc without setae.

Wing membrane clear, veins darker; 1 long basal costal seta, extending almost to humeral crossvein. Cell dm produced anteroapically. M_1_ and M_2_ with long stem vein proximal to M_1+2_ fork. CuA+CuP not visible. Pterostigma elongate, faint. Squama with setulae. Halter pale.

Legs brownish; fore femur with two stronger anterior setae on apical fourth; uniformly covered with rows of small dark setulae. All coxae with longer setae anteriorly; fore coxae with several erect setae. Fore and mid femora ventrally with some longer setulae on proximal half, some longer than width of segment.

Abdomen covered in small setae. Terminalia (Figs 6, 7): hypandrium subequal in length with epandrium; narrow, with 8 pairs of short setae. Epandrium subrectangular, covered with long setae especially ventrally and laterally; surstylus thumb-like on inner face apically. Clasping cercus unilobed, pointed apically; finger-like, with small basal projection on inner face with setae; fine on outer face near anterior margin and apex; inner face with stouter setae, especially near posterior margin. Phallus more or less linear, slender; distiphallus similar to phallus shaft, narrow, without swellings.

**Female.** Unknown.

##### Etymology.

The species is named after the Greek mythology character Iphigenia, the priestess of the Greek Goddess Artemis.

##### Remarks.

*Wiedemannia iphigeniae* sp. n. is known only from the type locality in Greece. The shape of the clasping cercus is similar to that of *W. aerea* Vaillant, 1967 (Pyrenees), but a distinct basal projection is lacking in the latter species.

#### Wiedemannia ljerkae

Taxon classificationAnimaliaDipteraEmpididae

Ivković & Sinclair sp. n.

http://zoobank.org/F9A07ACC-BB76-4D11-8736-FDD2414413B7

**Figures 2–5.**
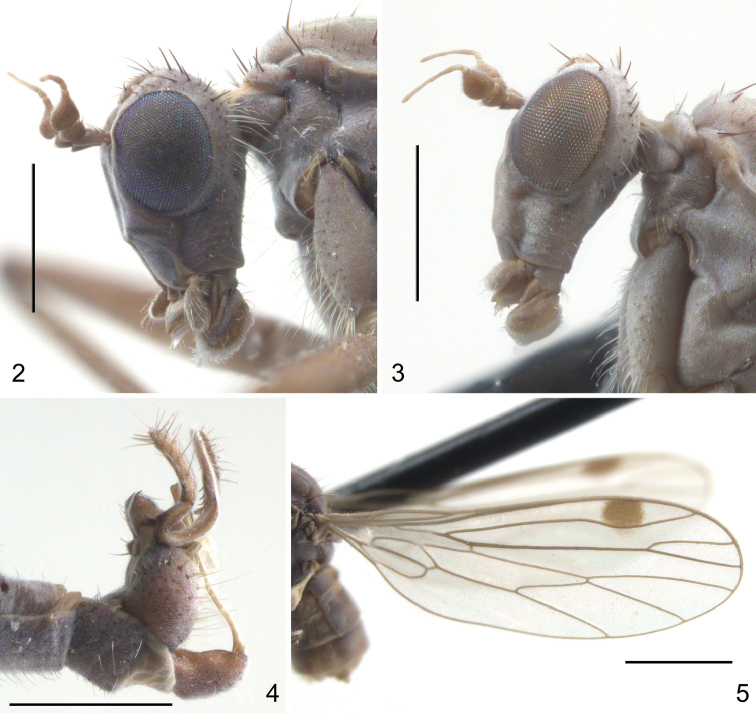
Heads, male terminalia and wing of *Wiedemannia* spp. **2** *W. ljerkae* Ivković & Sinclair, sp. n., male paratype, head, lateral view **3** *W. pseudoberthelemyi* Ivković & Sinclair, sp. n., male paratype, head, lateral view **4** *W. ljerkae* Ivković & Sinclair, sp. n., paratype, male terminalia, lateral view **5** *W. ljerkae* Ivković & Sinclair, sp. n., male paratype, wing, Scale bar: 0.5 mm (**2, 3, 4**); 1.0 mm (**5**).

Figs 1 , 2 , 4 , 5 , 8

##### Type locality.

Greece: Epirus, Igoumenitsa, River Thiamis, Soulopoulo, 39°32'00"N, 20°12'00"E.

##### Type material.

**Holotype** ♂ (in 80% ethanol), labelled: “HOLOTYPE/ *Wiedemannia*// *ljerkae* IVKOVIĆ et SINCLAIR// GREECE, Epirus,/ Igoumenitsa, R. Thiamis,/ Soulopoulo// 39°32'00"N, 20°12'00"E, 30.iv.1989,/ leg. B. Horvat, I. Sivec” (UZC). **Paratypes**: same data as holotype (2 ♂, 6 ♀, UZC; 3 ♂, 9 ♀, CNC (dried from alcohol); 2 ♂, 16 ♀, SMNH).

##### Additional material.

**GREECE**: Central Greece, Etolia, Peristera, Agrinio, 1 km S of Perkos, 300 m, 38°38'00"N, 21°45'00"E, 24.iv.1990 (SMNH); Peloponnese, Kato Klitoria, 450 m, 37°50'00"N, 22°10'00"E, 20.iv.1990 (SMNH); Peloponnisos, R. Kratis, 7 km N of Peristera, 600 m, 38°05'00"N, 22°14'00"E, 22.iv.1990 (SMNH); Peloponnese, Aroania Mts., Kastria, 21.iv.1990 (UZC).

##### Diagnosis.

This species of *Wiedemannia* is distinguished by the cercus with two long finger-like processes and a rounded pterostigma on the wings.

##### Description.

**Male.** Body length 3.5–4.5 mm, wing length 4.0–4.3 mm (colouration bleached by prolonged storage in alcohol). Head (Fig. 2) in lateral view higher than long; gena broad, more than half height of eye. Frons short, broader than face. Face wide, with distinct carina on lower margin, bare, lacking setae. Ocellar setae short and fine; one pair of vertical setae; about 7–8 distinct upper postocular setae; lower postocular setae finer and merging with longer setae on middle and lower occiput; numerous dark setulae on vertex and between ocellar area and eye margin. Antenna brown; postpedicel and stylus minutely pubescent; pedicel slightly shorter than half length of scape, with complete circlet of subapical setae; postpedicel apically pointed, stylus nearly twice length of postpedicel; scape with setulae dorsally.

Mesonotum with 5 pairs of dorsocentral setae with short setulae interspersed. Acrostichal setae small and fine, biserial, extended onto prescutellar depression; 1 strong postpronotal seta and 2–4 small setulae; 2 notopleural setae with several short setulae; 1 presutural supra-alar seta and many small anterior setulae; 1–2 postsutural supra-alar setulae; 1 postalar seta. Antepronotum with 3–4 pairs of dark, strong setae and some smaller setae. Proepisternum with some fine, long setae. Katepisternum with some short setulae on posterior margin. Laterotergite with fine, pale setae. One pair of strong marginal scutellar setae, with many scattered setulae on disc.

Wing (Fig. 5) membrane infuscate, veins darker; 1 long basal costal seta extending almost to humeral crossvein. R_2+3_ dipped beneath pterostigma. Cell dm produced anteroapically. M_1_ and M_2_ originating separately, together or sometimes with a very short stem vein proximal to M_1+2_ fork. CuA+CuP appearing as crease. Pterostigma broad, circular to squarish, dark brown, extending faintly beyond R_2+3_. Squama with setulae. Halter pale, yellowish.

Legs mostly brown; fore femur with one stronger preapical anterior seta; uniformly covered with rows of small dark setulae. All coxae with longer setae anteriorly. Fore and mid femora ventrally with some longer setulae on proximal half.

Abdomen concolourous with thorax, covered in short setae. Terminalia (Figs 4, 8): hypandrium shorter than epandrium, bearing 4 pairs of setae. Epandrium subquadrate, covered with long dark setae especially ventrally and laterally; surstylus slender, digitiform. Clasping cercus with two long, slender, finger-like processes and small basal lobe with crown of spine-like setae on inner face apically; finger-like lobes with long setae distally; posterior finger-like lobe with inner cluster of stout setae at mid-length. Phallus more or less linear, very slender; distiphallus with distinct swelling at mid-length.

**Female.** Similar to male except pterostigma smaller, more elliptical, not extending beyond R_2+3_; cercus short, ovate and minutely pilose.

##### Etymology.

The species is named after the first author’s mother, Katica Ljerka Ivković, for all those things that mothers do for all of us.

##### Remarks.

*Wiedemannia ljerkae* sp. n. is known only from several localities in Greece. On the basis of the multiple slender lobes of the clasping cercus and distinct pterostigma, *W. ljerkae* sp. n. appears closely related to *W. braueri* (Mik, 1880) and *W. tricuspidata* (Bezzi, 1905) (see Engel 1918, 1940).

#### Wiedemannia nebulosa

Taxon classificationAnimaliaDipteraEmpididae

Ivković & Sinclair sp. n.

http://zoobank.org/61BC89BA-016F-43BC-A59F-BA5B5259EAD9

Figs 1 , 9

##### Type locality.

Greece: Thrace, north of Dipotama, 41°24'24"N, 24°37'19"E, 1400 m.

##### Type material.

**Holotype** ♂, labelled: “GREECE: Thrace/ N of Dipotama/ 41°24'24"N, 24°37'19"E/ 23.v.1994; 1400 m/ leg. B. Horvat, I. Sivec”; “HOLOTYPE/ *Wiedemannia*/ *nebulosa*/ Ivković & Sinclair” (CNC, dried from alcohol). **Paratypes**: same data as holotype (1 ♂, 1 ♀, CNC, dried from alcohol).

##### Diagnosis.

This species of *Wiedemannia* is distinguished by the faint clouding about crossveins and base of radial fork, shape of the clasping cercus and position of distiphallus on the phallic shaft.

##### Description.

**Male.** Body length 3.8–4.5 mm, wing length 5.2–5.3 mm (colouration slightly bleached by prolonged storage in alcohol). Head dark with brown frons and vertex, remainder of head with blue pruinescence; head higher than long; gena narrow, one-quarter height of eye. Frons short, broader than face. Face wide, with distinct carina on lower margin, bare, lacking setae. One pair of long ocellar setae and one pair of vertical setae; 6–7 distinct upper postoculars; lower postocular setae finer and merging with longer setae on middle and lower occiput; a few small setulae present on vertex and in ocellar area. Antenna brown; postpedicel and stylus minutely pubescent; scape longer than pedicel, with setulae dorsally; pedicel with complete circlet of apical setae; postpedicel apically pointed; stylus twice length of postpedicel.

Scutum dark brown with pair of faint black vittae between dorsocentral row and acrostichals and bluish stripe medially; prescutellar depression with blue pruinescence. Pleura clothed with blue pruinescence. Mesonotum with 5 pairs of dorsocentral setae without short setulae interspersed. Acrostichal setae short and fine, biserial, extending onto prescutellar depression; 1 strong postpronotal seta; 2 notopleural setae and several short setae; 1 presutural supra-alar seta and several small anterior setulae; 1 postalar seta. Antepronotum with 1 pair of strong setae. Proepisternum with some fine setulae. Katepisternum without setulae. Laterotergite with fine, pale setae. One pair of strong marginal scutellar setae; disc bare.

Wing membrane infuscate with darkening at apex of cell dm, radial fork and r-m crossvein; veins darker; 1 short basal costal seta ending before humeral crossvein. Cell dm produced anteroapically. M_1_ and M_2_ originating separately from cell dm. CuA+CuP in form of short streak. Pterostigma broad and elongate, very distinct. Squama with setulae. Halter yellowish brown.

Legs mostly brown; fore femur with 2–3 strong anterior setae on apical quarter; uniformly covered with rows of small dark setulae. All coxae with longer setae anteriorly; fore coxa with 1–2 erect setae. Fore and mid femora ventrally with some longer setulae on proximal half.

Abdomen concolourous with thorax, covered in short setae. Pruinescence darker on tergites than sternites. Terminalia (Fig. 9): hypandrium subequal in length with epandrium, with 5 pairs of setae. Epandrium irregularly subquadrate, with several stouter and longer setae (shown by enlarged sockets) in addition to normal setae ventrally and laterally; surstylus short, digitiform with rounded apex; subepandrial sclerite projecting slightly beyond epandrium near surstylus. Clasping cercus pale brown, broad, gradually tapered to rounded apex; inner posterior margin with long peg-like setae. Phallus more or less linear, slender; distiphallus without swelling at mid-length; distiphallus with serrate membranous margin, extending onto shaft.

**Female.** Similar to male. Terminalia: cercus short ovate and minutely pilose.

##### Etymology.

The species name is derived from the Latin *nebulosus* (misty, cloudy, dark), in reference to the clouding about the crossveins.

##### Remarks.

*Wiedemannia nebulosa* sp. n. is known only from the type locality in Greece. On the basis of the shape of the clasping cercus, this new species is similar to *W. carpathica* Vaillant, 1967 (eastern Carpathians), *W. pyrenaica* Vaillant, 1967 (Pyrenees) and perhaps *W. wachtli* (Mik, 1880).

#### Wiedemannia pseudoberthelemyi

Taxon classificationAnimaliaDipteraEmpididae

Ivković & Sinclair sp. n.

http://zoobank.org/BD1AFAB9-06BD-4BEC-A08F-F3D80E8FAFE0

Figs 1 , 3 , 10

##### Type locality.

Greece: Etolia, River Mornos, Nafpaktos, 38°23'N, 21°51'E.

##### Type material.

**Holotype** ♂ (in 80% ethanol), labelled: “HOLOTYPE/ *Wiedemannia*// *pseudoberthelemyi* IVKOVIĆ// et SINCLAIR/, GREECE, Etolia,// River Mornos,/ Nafpaktos,/ 38°23'N, 21°51'E, 23.iv.1990,// leg. B. Horvat, I. Sivec” (UZC). **Paratypes**: same data as holotype (1 ♂, 3 ♀, UZC; 3 ♂, 6 ♀, CNC, dried from alcohol).

##### Additional material.

**GREECE**: Central Greece, Panaitoliko Mts., R. Tavropos, Kalesmeno, 300 m, 38°56'N, 21°40'E, 29.iv.1989 (SMNH); Central Greece, Etolia, Agrinio, Agia Soufia, 100 m, 38°36'N, 21°26'E, 24.iv.1990 (SMNH); Etolia, Vardousia Mts., R. Evinos, Grammeni Oxia, 800 m, 38°43'N, 22°00'E, 28.iv.1990 (SMNH).

##### Diagnosis.

This species of *Wiedemannia* is distinguished by the long gena and the mitten-shaped clasping cercus, which is extremely similar to that of Wiedemannia (Chamaedipsia) berthelemyi Vaillant & Vinçon, 1987.

##### Description.

**Male.** Body length 3.5–4.1 mm, wing length 3.5–3.6 mm (colouration bleached by prolonged storage in alcohol). Head (Fig. 3) in lateral view higher than long; gena broad, three-quarters height of eye. Frons short, broader than face. Face wide, with distinct carina on lower margin, bare, lacking setae. One pair of short ocellar setae and one pair of vertical setae; about 6 distinct upper postocular setae; lower postocular setae finer and merging with longer setae on middle and lower occiput; few small setulae present on vertex and between ocellar area. Antenna brown; postpedicel and stylus minutely pubescent; scape longer than pedicel, with setulae dorsally; pedicel with complete circlet of apical setae; postpedicel apically pointed; stylus nearly twice length of postpedicel.

Mesonotum with 5 pairs of dorsocentral setae with short setulae interspersed. Acrostichal setae short and fine, biserial, extending to prescutellar depression; 1 strong postpronotal seta; 2–3 notopleural setae and several short setae of variable size; 1 presutural supra-alar seta and numerous small setulae; 1 postalar seta. Antepronotum with 1 pair of strong setae and a few shorter setae. Proepisternum with some fine setulae. Katepisternum with a few (1–3) setulae. Laterotergite with fine, pale setae. One pair of strong marginal scutellar setae with many scattered setae on disc.

Wing membrane infuscate, veins darker; 1 short basal costal seta ending before humeral crossvein. Cell dm produced anteroapically. Veins M_1_ and M_2_ originating together with short stem vein proximal to M_1+2_ fork. Vein CuA+CuP extremely faint. Pterostigma elongate, indistinct. Squama with setulae. Halter yellowish.

Legs mostly brown; fore femur with 1 strong preapical anterior seta; uniformly covered with rows of small dark setulae. All coxae with longer setae anteriorly. Fore and mid femora ventrally with some longer setulae on proximal half.

Abdomen concolourous with thorax, covered in short setae. Pubescence darker on tergites than sternites. Terminalia (Fig. 10): hypandrium subequal in length with epandrium, with 6 pairs of setae. Epandrium irregularly subquadrate, with 2–3 stouter and longer setae (shown by enlarged sockets) in addition to regular setae ventrally and laterally; surstylus very slender, hook-shaped. Clasping cercus yellowish-brown, broad, mitten-shaped, with thumb-like anterior lobe; posterior lobe truncate apically; thumb-like lobe with long outer setae; stout setae with multi-branched apex covering most of inner face of cercus. Phallus more or less linear, slender; distiphallus with swelling at mid-length.

**Female.** Similar to male. Terminalia: cercus short ovate and minutely pilose.

##### Etymology.

The species name is derived from the name Wiedemannia (Chamaedipsia) berthelemyi because of the similarity of the clasping cercus with that of this species.

##### Remarks.

*Wiedemannia pseudoberthelemyi* sp. n. is known only from parts of Greece. This new species differs from *W. berthelemyi* on the basis of the truncate posterior lobe of the clasping cercus (pointed in *W. berthelemyi*) and in having only a single preapical seta on the fore femur (2 in *W. berthelemyi*). The odd stout setae with multi-branched tips on the inner face of the clasping cercus were not noted by Vaillant and Vinçon (1987) and the absence of the swelling on the distiphallus (Vaillant and Vinçon 1987, fig. 32) is likely an artefact caused by the acid clearing process. The holotype of *W. berthelemyi* was not examined.

Additional similar species that could be included in this group based on the shape of the clasping cercus include: *W. angelieri* Vaillant, 1967 (Pyrenees), *W. vedranae* Ivković & Sinclair, 2014 (Sierra Nevada, Spain), and *W. queyrasiana* Vaillant, 1956 (European Alps).

### Hemerodromiinae

#### Chelifera horvati

Taxon classificationAnimaliaDipteraEmpididae

Ivković & Sinclair sp. n.

http://zoobank.org/9DE403F2-5A28-4E6F-A485-A4D42308165D

Figs 1 , 11

##### Type locality.

Greece: Central Greece, Etolia, Arta, Loutraki.

##### Type material.

**HOLOTYPE** ♂, labelled: “GREECE: Central Greece/ Etolia, Arta, Loutraki/ 16.iv.1990/ leg. B. Horvat, I. Sivec”; “HOLOTYPE/ *Chelifera*/ *horvati*/ Ivković & Sinclair” (CNC, dried from alcohol).

##### Diagnosis.

A yellow-brown species with distinct, brown and rounded pterostigma, characterized in the male by dark brown cercus with elongate, slender forked process at mid-length, posteriorly tapered epandrium with stout inner setae and membranous distiphallus with two elongate lobes.

##### Description.

**Male.** Body length 4 mm, wing length 3.6 mm. Head dorsoventrally flattened, dark brown; ocellar triangle dark brown; all setae whitish. Eyes iridescent black; narrowly separated on face. Face with thick, whitish pubescence. One pair of postocular setae and scattered fine setae on vertex. Occiput bearing scattered fine setulae; gena with rather dense short, downwardly directed whitish pile. Antenna whitish, with scape and pedicel bearing distinct short dorsal setulae; postpedicel about 1.5× as long as wide, stylus much shorter than postpedicel.

Thorax elongate; yellow, all setae yellowish. Mesonotum with pair of brown vittae, extending around prescutellar depression; small dark spot posterior to postpronotal lobe and larger dark spot near wing base. Holotype missing most thoracic setae.

Wing (slightly damaged) membrane transparent, veins yellow; pterostigma dark, rounded, with R_2+3_ arched around it; fork of R_4+5_ less than 90°; cell r_4_ rather long, R_5_ nearly 2× as long as R_4_. Halter pale.

Legs whitish yellow, apical two tarsal segments on all legs brown. Fore coxa about 8× longer than wide with several pale dorsoapical setae. Fore femur slightly longer than fore coxa, more than 4× longer than wide, evenly inflated, widest at middle. Fore femur with two rows of black ventral denticles and two rows of strong outer brownish-yellow ventral setae, with following chaetotaxy: 20 anteroventral denticles, 6 anteroventral spine-like setae, 21 posteroventral denticles, 6 posteroventral spine-like setae; denticles closely spaced and rows converging distally; posteroventral spine-like setae shorter distally. Fore tibia 0.6× as long as fore femur, evenly curved with anteroventral row of short, spine-like setae; with apicoventral dark spur-like seta, longer than width of tibia. Mid and hind femora with anteroventral row of short, slender setae.

Abdomen yellow ventrally, brown dorsally, with pale setae most conspicuous on hind margin of posterior sternites. Terminalia (Fig. 11): cercus dark brown, thick, with narrow, elongate process at mid-length with forked apex (process folded horizontally in non-macerated condition); anterior end of cercus pointed and curved medially, with long setae, posterior end of cercus rounded; cercus wider then epandrium. Epandrium yellowish-brown, concave medially, posteriorly pointed with 5 stout setae on inner apical margin directed medially; entire epandrium covered in numerous setae. Hypandrium yellow, quadrate, with posteroapical lobe and concave posterior margin; pale setae on posteroventral face. Postgonite slender, sickle-shaped. Distiphallus membranous, expanded into two elongate lobes; apex of posterior lobe with pigmented arch-shaped sclerotization.

**Female.** Unknown.

##### Etymology.

The new species is named after the late Dr Bogdan Horvat, mentor of the first author, colleague and during his life a leading expert on the genus *Chelifera* Macquart.

##### Remarks.

*Chelifera horvati* sp. n. is known only from one site in Greece. The narrow pigmented and sclerotized apex of the distiphallus of *C. horvati* sp. n. is similar in *C. concinnicauda* Collin, 1927, *C. diversicauda* Collin, 1927, *C. giraudae* Vaillant, 1982 and *C. subangusta* Collin, 1961 (see Collin 1961 and Vaillant 1982).

### Key to species of aquatic Empididae of Greece

(written primarily for male specimens; some couplets modified from Collin (1961) and Vaillant (1982); Wiedemannia (Philolutra) hygrobia (Loew) is included in the key, although Greek records not confirmed)

**Table:** 

1	Fore femur with one or two rows of black, peg-like setae ventrally; fore femur width 2–3 times that of fore tibia	**Hemerodromiinae**...**2**
–	Fore femur without black, peg-like setae ventrally; fore femur width less than 1.5 times that of fore tibia	**Clinocerinae**...**11**
2	Cell cua (anal cell) and crossvein bm-cu absent (discal cell absent); R_1_ meeting costa before middle of wing	***Hemerodromia* Meigen**...**3**
–	Cells cua (anal cell) and dm present (crossvein bm-cu present); R_1_ meeting costa beyond middle of wing	***Chelifera* Macquart**...**5**
3	Scutum with dark spot posterior to postpronotal lobe; cercus inflated, kidney-shaped in dorsal view (Collin 1961, fig. 302)	***Hemerodromia melangyna* Collin**
–	Scutum without dark spot posterior to postpronotal lobe; cercus not inflated	**4**
4	Cercus with shallow, semi-circular apical excision (Collin 1961, fig. 299)	***Hemerodromia oratoria* (Fallén)**
–	Cercus without apical excision, apex rounded with short, stout inner setae (Collin 1961, fig. 300a)	***Hemerodromia unilineata* Zetterstedt**
5	Pterostigma very indistinct, long ovate and pale yellow (male terminalia: Collin 1961, fig. 295)	***Chelifera trapezina* (Zetterstedt)**
–	Pterostigma distinct, circular and black or brownish	**6**
6	Male cercus simple, without lobe in lateral view	**7**
–	Male cercus with lobe in lateral view	**9**
7	Male cercus small and thin, narrower than epandrium (Collin 1961, fig. 292)	***Chelifera angusta* Collin**
–	Male cercus as broad as epandrium	**8**
8	Male cercus, viewed from above, with a distinct projection near middle of inner edge (Collin 1961, fig. 287)	***Chelifera precabunda* Collin**
–	Male cercus, viewed from above, without a distinct projection near middle of inner edge (Collin 1961, fig. 286)	***Chelifera precatoria* (Fallén)**
9	Male cercus with narrow, elongate dorsal process at mid-length, with forked apex (Fig. 11)	***Chelifera horvati* sp. n.**
–	Male cercus without dorsal process at mid-length	**10**
10	Posterior lobe of male cercus arched; hypandrium in lateral view tapered and narrowed posteriorly (Vaillant 1982, figs 5g, 7a)	***Chelifera stigmatica* (Schiner)**
–	Posterior lobe of male cercus conical; hypandrium in lateral view rouned and broadly prolonged posteriorly (Vaillant 1982, figs 5i, 9a)	***Chelifera barbarica* Vaillant**
11	Neck arising high on occiput, from near top of head	***Dolichocephala* Macquart**...**12**
–	Neck arising near centre of occiput or level with centre of eye	**16**
12	Wings without white rounded spots or irrorations, at most only faint white streaks in cells	***Dolichocephala guttata* (Haliday)**
–	Wings with white rounded spots or irrorations	**13**
13	Wings with only white streak in cell r_2+3_ (proximal section) (Wagner 1995, fig. 4); clasping cercus elongate and straight, narrow on apical half (Wagner 1995, fig. 10)	***Dolichocephala cretica* Wagner**
–	Wings with pair of white spots in cell r_2+3_ (proximal section) (Wagner 1995, figs 3, 6); clasping cercus arched	**14**
14	Surstylus unforked; clasping cercus strongly arched (Engel 1939, fig. 46)	***Dolichocephala ocellata* (Curtis)**
–	Surstylus forked (Wagner 1995, figs 8, 13); clasping cercus gradually arched	**15**
15	Clasping cercus L-shaped, with peg-like seta at inner apex (Wagner 1995, fig. 7)	***Dolichocephala vaillanti* Wagner**
–	Clasping cercus arched medially, with peg-like seta subapically (Wagner 1995, fig. 12)	***Dolichocephala zwicki* Wagner**
16	Tarsomeres 2–4 of foreleg subequal in length	**17**
–	Tarsomere 2 of foreleg much longer than tarsomeres 3 or 4, often twice length of tarsomere 4	**18**
17	Proboscis as long as head; labrum long and slender; labellum not sucker-like (Wagner 1981, fig. 8)	***Roederiodes malickyi* Wagner**
–	Proboscis shorter than head; labrum subtriangular; labellum sucker-like (Engel 1939, text fig. 92)	***Clinocerella siveci*** (**Wagner & Horvat)**
18	Lower margin of face lacking notch or deep cleft above mouthparts; apical phallus filament not articulated	***Clinocera* Meigen**...**19**
–	Lower margin of face with notch or deep cleft; apical phallic filament articulated	**21**
19	Comb of preapical anterior setae on fore femur absent; postpronotal seta reduced, shorter and thinner than notopleural setae; postsutural supra-alar setae absent	***Clinocera nigra* Meigen**
–	Comb of preapical anterior setae on fore femur present; postpronotal seta well developed, similar to scutal setae; postsutural supra-alar setae present	**20**
20	Wings with faint clouding about crossveins; apex of femora (“knees”) light brown, compared to bluish pruinescent femur; surstylus elongate, apex rounded (Collin 1961, fig. 311b)	***Clinocera stagnalis* (Haliday)**
–	Wings without clouding; apex of femora not paler than remaining femur; surstylus subtriangular, with narrow anterior apex (Fig. 12)	***Clinocera megalatlantica* (Vaillant)**
21	Face with setulae along inner margin of eye	***Kowarzia* Mik**...**22**
–	Face bare, without setulae along inner margin of eye	**25**
22	Coxae and femora dark, brown (male terminalia: Vaillant 1965, figs 3e, f)	***Kowarzia plectrum* (Mik)**
–	Coxae and femora pale, yellowish	**23**
23	Surstylus deeply forked (Collin 1961, fig. 314c)	***Kowarzia bipunctata* (Haliday)**
–	Surstylus unforked	**24**
24	Surstylus as broad as clasping cercus; clasping cercus digitiform, strongly curved at middle, generally similar in width until apex (Vaillant 1965, figs 3b, d)	***Kowarzia madicola* (Vaillant)**
–	Surstylus long and slender, much thinner than clasping cercus; clasping cercus broad, gently curved (Collin 1961, fig. 314a)	***Kowarzia barbatula* Mik**
25	Wings with distinct spots; pterostigma clearly outlined, elliptical	***Phaeobalia dimidiata* (Loew)**
–	Wings lacking spots; pterostigma usually either both faint and elongate or dark and circular	***Wiedemannia* Zetterstedt**...**26**
26	Pterostigma rounded, usually very distinct and large (Fig. 5)	**27**
–	Pterostigma elongate and narrow, often indistinct	**35**
27	Gena width more than half vertical diameter of eye (Fig. 2)	**28**
–	Gena width less than half vertical diameter of eye	**34**
28	Clasping cercus deeply divided into 2 or 3 elongate finger-like lobes (Fig. 4)	**29**
–	Clasping cercus not divided into elongate finger-like lobes	**30**
29	Clasping cercus with 2 elongate lobes and shorter, broad anterior lobe bearing peg-like apical setae (Fig. 8)	***Wiedemannia ljerkae* sp. n.**
–	Clasping cercus with 3 elongate lobes, anterior lobe narrower and slightly shorter than posterior lobes, without peg-like setae (Engel 1940, fig. 95)	**Wiedemannia (Wiedemannia) tricuspidata (Bezzi)**
30	Base of clasping cercus prolonged anteriorly, arched around epandrium	**31**
–	Base of clasping cercus not prolonged anteriorly around epandrium	**33**
31	Apex of clasping cercus tapered and narrow (Engel 1940, fig. 88)	**Wiedemannia (Wiedemannia) bilobata Oldenberg**
31	Apex of clasping cercus bluntly rounded and broad	**32**
32	Apex of clasping cercus prolonged; base of clasping cercus with stout setae confined to upper inner edge (Vaillant and Wagner 1990, figs 1, 2)	**Wiedemannia (Wiedemannia) graeca Vaillant & Wagner**
–	Apex of clasping cercus slightly expanded, not prolonged anteriorly; base of clasping cercus with stout setae covering most of inner face (Wagner 1990, figs 3, 4)	**Wiedemannia (Wiedemannia) dyonysica Wagner**
33	Clasping cercus with posterior margin deeply invaginated, forming pair of narrow basal lobes (Joost 1982, figs 1, 2)	**Wiedemannia (Wiedemannia) andreevi Joost**
–	Clasping cercus L-shaped (Engel 1940, fig. 91)	**Wiedemannia (Wiedemannia) dinarica Engel**
34	Clasping cercus broad in lateral view, expanded at mid-length (Engel 1940, fig. 97)	**Wiedemannia (Pseudowiedemannia) lamellata (Loew)**
–	Clasping cercus very narrow on basal half in lateral view, with short lobe at mid-length directed medially (Engel 1940, fig. 98)	**Wiedemannia (Pseudowiedemannia) microstigma (Bezzi)**
35	All femora with distinct anterior and posterior preapical setae (male terminalia: Engel 1940, fig. 78)	**Wiedemannia (Eucelidia) zetterstedti (Fallén)**
–	Femora without distinct preapical setae, or at least only anterior seta present on fore femur	**36**
36	Acrostichals only present anterior to second dorsocentral seta (male terminalia: Engel 1940, fig. 79)	**Wiedemannia (Roederella) czernyi (Bezzi)**
–	Acrostichals extending to at least prescutellar depression	**37**
37	Gena width more than half vertical diameter of eye (Fig. 3)	***Wiedemannia pseudoberthelemyi* sp. n.**
–	Gena width less than half vertical diameter of eye	**38**
38	Clasping cercus short and broad, height and width of lobe subequal	**39**
–	Clasping cercus prolonged dorsally, distinctly higher than wide	**43**
39	Clasping cercus circular, without lobes (Mandaron 1964, figs B, E)	**Wiedemannia (Chamaedipsia) aequilobata Mandaron**
39	Clasping cercus bilobed, mitten-shaped	**40**
40	Anterior lobe of clasping cercus narrow, thumb-like (Vaillant 1967, figs 2.3, 2.4)	**Wiedemannia (Philolutra) angelieri Vaillant**
–	Anterior lobe of clasping cercus broad, subequal in width to posterior lobe or small, not longer than wide	**41**
41	Anterior lobe of clasping cercus broad, subequal in width to posterior lobe (Joost 1981, figs 7, 8)	**Wiedemannia (Philolutra) chvali Joost**
–	Anterior lobe of clasping cercus small, not longer than wide	**42**
42	Clasping cercus without long setae anteriorly at base (Wagner 1981, figs 5, 6)	**Wiedemannia (Chamaedipsia) ariadne Wagner**
–	Clasping cercus with long setae anteriorly at base (Engel 1940, fig. 102)	**Wiedemannia (Philolutra) hygrobia (Loew)**
43	Clasping cercus mitten-shaped, with thumb-like anterior lobe (Engel 1940, fig. 101)	**Wiedemannia (Philolutra) fallaciosa (Loew)**
–	Clasping cercus not mitten-shaped	**44**
44	Fore femur with a single distinct anterior seta at about one-sixth from apex	**45**
–	Fore femur without setae or with several distinct anterior setae at about one-sixth from apex	**46**
45	Clasping cercus long, slender and parallel-sided, yellow, nearly twice as long as width of epandrium (Collin 1961, fig. 313c; Engel 1940, fig. 86)	**Wiedemannia (Chamaedipsia) lota Walker**
–	Clasping cercus slightly longer than width of epandrium and tapered apically with narrow basal lobe-like expansion (Fig. 6)	***Wiedemannia iphigeniae* sp. n.**
46	Clasping cercus with broad base, bent at nearly right angles, L-shaped (Ivković et al. 2012, figs 2, 3)	***Wiedemannia artemisa* Ivković & Plant**
–	Clasping cercus with broad base and gradually tapered apically	**47**
47	Phallus shaft extended well beyond base of distiphallus; clasping cercus with stout, blunt-tipped setae along inner posterior margin (Fig. 9); wing with faint clouding about crossveins	***Wiedemannia nebulosa* sp. n.**
–	Phallus shaft not extended beyond base of distiphallus; clasping cercus with long thick setae along inner margin (Vaillant 1967, fig. 2.9); wing without faint clouding about crossveins	**Wiedemannia (Chamaedipsia) beckeri (Mik)**

**Figures 6–8.**
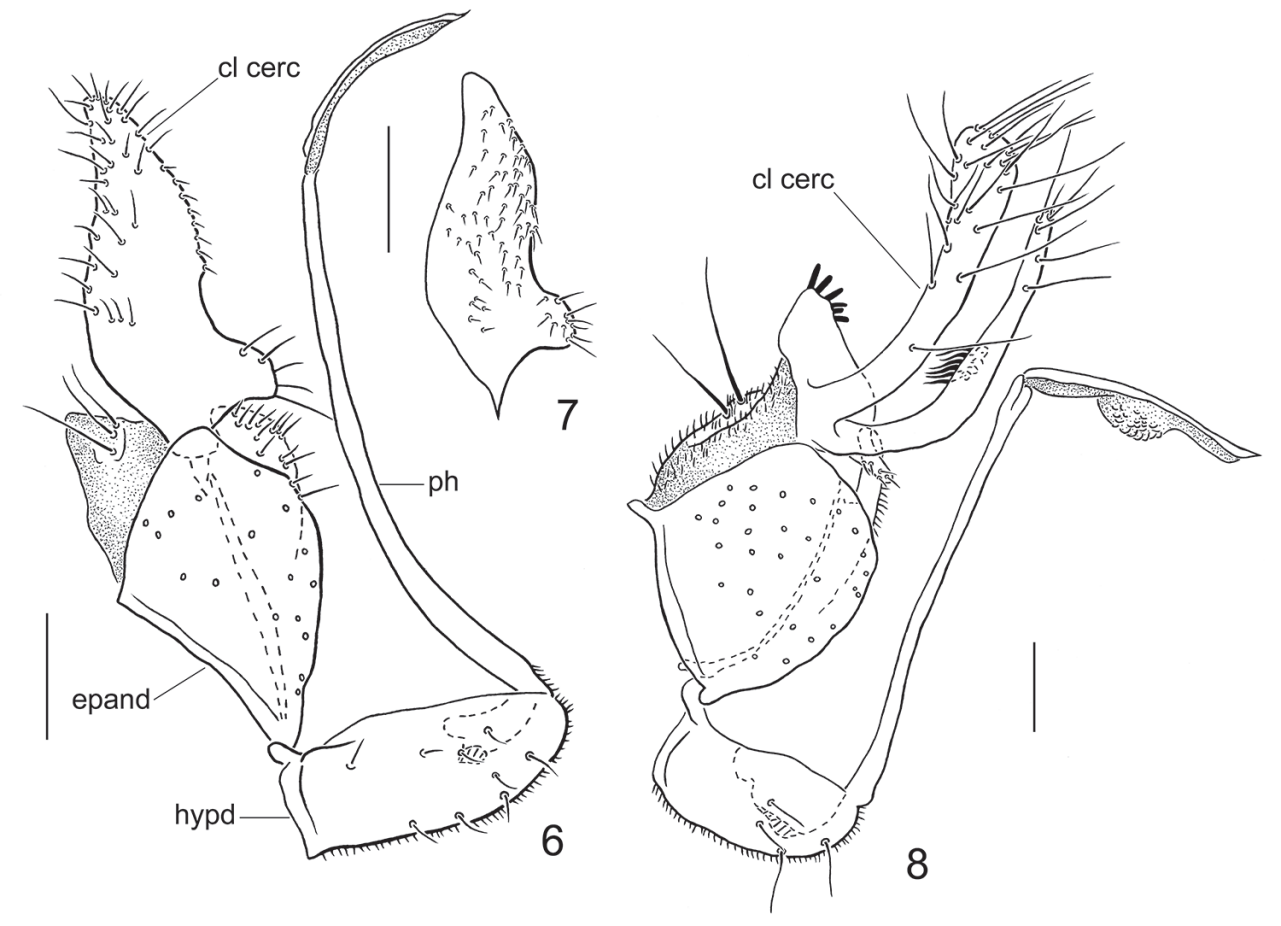
Male terminalia of *Wiedemannia* spp., lateral view. **6** *W. iphigeniae* Ivković & Sinclair, sp. n., holotype **7** *W. iphigeniae* Ivković & Sinclair, sp. n., holotype, clasping cercus, inner view **8** *W. ljerkae* Ivković & Sinclair, sp. n., paratype.

### List of Empididae of Greece (Clinocerinae & Hemerodromiinae)

The following format is used for the distributional data: Literature references – name of the site and in brackets the reference citation and site ID; New records – name of the site and in brackets the site ID. All the sites and their numbers are listed in Table 1.

### Subfamily Clinocerinae

***Clinocera megalatlantica* (Vaillant, 1957)**

**New records.** Thrace, Samothrace, hygropetric zone of stream at the church of Kreminotissa (15).

**Remarks.** This species is newly recorded from Greece. The male terminalia of this species are illustrated (Fig. 12) to highlight additional detail not shown in the original drawing of Vaillant (1957, fig. IIC).

***Clinocera nigra* Meigen, 1804**

**New records.** Macedonia, Pieria Mts. 2 (52); Epirus, R. Aheron, N of Gliki (115); Central Greece, Etolia, Vardousia Mts., 13 km S of Gardiki (164); Central Greece, Etolia, Panaitoliko Mts., Klepa (170); Central Greece, Etolia, Nafpaktos, Anthofito (174); Central Greece, Etolia, Agrinio, R. Evinos, Kato Hrisovitsa, Diasellaki (183); Central Greece, Etolia, Agrinio, Agia Soufia (191); Central Greece, Etolia, Nafpaktos, Koutsopanneika (203); Peloponnese, tributary of R. Krathis, 7 km N of Peristera (215); Peloponnese, 2 km N of Peristera (216); Peloponnese, Ano Potames, Kalivitis (219).

***Clinocera stagnalis* (Haliday, 1833)**

**Literature references.** Macedonia, Grevena, stream S of R. Aliakmon by Kamilas Pigi (Wagner 1995) (58); Macedonia, Vernon, influx of Aliakmon between Gavros and Aposkepos (Wagner 1995) (61); Macedonia, Chalkidiki, Chlomon Oros., valley on the southern slope (Wagner 1995) (68); Epirus, Pindus Mts., Metsovo, meadow source easthang (Wagner 1995) (94); Epirus, Xerovouni Mts., Plaka, R. Arachthos, u. Agnatha (Wagner 1995) (105).

**New records.** Thrace, N of Xanthi (18); Thrace, N of Dipotama, 1 (19); Thrace, N of Dipotama 3 (22); Thrace, Dit. Rodopi, Skaloti (29); Thrace, Dit. Rodopi 1 (30); Thrace, Dit. Rodopi, E of Mikromilia (35); Macedonia, Dit. Rodopi, Elatia forest (37); Macedonia, E of Mikroklisoura (38); Macedonia, N of Stavros (39); Macedonia, N of Agios Dimitrios (43); Macedonia, Pieria Mts., S of Elatohori (44); Macedonia, Pieria Mts., E of Fteri (46); Macedonia, Pieria Mts., Fteri (47); Macedonia, Pieria Mts., W of Fteri (48); Macedonia, Pieria Mts., E of Velventos (50); Macedonia, Pieria Mts., 1 (51); Macedonia, Pieria Mts. 2 (52); Macedonia, Pieria Mts., 3 (53); Macedonia, E of Velventos (54); Macedonia, Phalacro Mts., N of Livadero (55); Macedonia, Grevena, Milea (56); Macedonia, Grevena, 6 km S of Milea (57); Macedonia, Kastoria, Nestorio (62); Thessaly, Pieria Mts., S of Livadi (74); Thessaly, 5 km W of Palea Giannitsou (75); Thessaly, Deskati (76); Thessaly, S of Asprokklisia (78); Thessaly, Kalambaka, Agios Nikolaos (80); Thessaly, Trikala, Stournareika (81); Thessaly, Trikala, Kato Palagokaria (82); Thessaly, Trikala, 9 km S of Chrisomilea (90); Epirus, Metsovo, Katara Pass (96); Epirus, Metsovo, R. Metsovitikos (99); Epirus, Metsovo, Lakmos Mts., Anthohori, (bellow rapid river) (100); Epirus, Metsovo, Lakmos Mts., Anilio (15 km S influx) (102); Epirus, Metsovo, 14 km W of Milea (103); Epirus, Ioannina, R. Zagoritikos, Karies (106); Epirus, 10 km N of Louros (110); Epirus, Ioannina, R. Voidomatis, Aristi (112); Central Greece, Etolia, Lamia, Ieraklia (145); Central Greece, Oeta Mts., between Kastanea and Katafygio (147); Central Greece, Etolia, Vardousia Mts., 5 km N of Grammeni Oxia (153); Central Greece, Etolia, Vardousia Mts., R. Evinos, Grammeni Oxia (154); Central Greece, Etolia, Vardousia Mts., 9 km N of Grammeni Oxia (155); Central Greece, Etolia, Vardousia Mts., 7 km N of Grammeni Oxia (156); Central Greece, Etolia, Vardousia Mts., 2 km W of Gardiki (166); Central Greece, Tymfristos Mts., R. Sperhios, Lamia (168); Central Greece, Etolia, Panaitoliko Mts., Klepa (170); Central Greece, Karpenisi, Agios Nikolaos (175); Central Greece, Etolia, Nafpaktos, tributory of R. Evinos, 6 km N of Pokista (176); Central Greece, Etolia, Lamia, Pavliani (192); Central Greece, Etolia, Agrinio, Panaitoliko Mts., Palagohori (199); Central Greece, Etolia, Nafpaktos, Koutsopanneika (203); Peloponnese, R. Krathis, 7 km N of Peristera (214); Peloponnese, tributary of R. Krathis, 7 km N of Peristera (215); Peloponnese, R. Krathis, Peristera (217); Peloponnese, Aroania Mts., Zarouhla (220); Peloponnese, Aroania Mts., Xelmos (bellow), Valtos, Zarelia (221); Peloponnese, Pagrati (225); Peloponnese, Aroania Mts., Kastria (226); Peloponnese, Kato Klitoria (227); Peloponnese, Labia Mts., Amigdalia (229); Peloponnese, R. Piro, Elliniko (230); Peloponnese, Panachaiko Mts., tributory of R. Selinous, Leontio (231); Peloponnese, Erymanthos Mts., Lechouri (233); Peloponnese, Panachaiko Mts., Veteika (234); Peloponnese, Erymanthos Mts., Kato Vlasia (235); Peloponnese, Panachaiko Mts., Kounaveika (near village) (236); Peloponnese, Erymanthos Mts., Profitis Ilias (239); Peloponnese, Erymanthos Mts., Stavrohori, Eliniko (241); Peloponnese, Erymanthos Mts., S of Spartia (243); Peloponnese, Stavrodromi (245); Peloponnese, Abelokipi (246); Peloponnese, Panachaiko Mts., Kristalovrisi (stream) (248).

***Clinocerella siveci* (Wagner & Horvat, 1993)**

**Literature references.** Central Greece, Etolia, Panaitoliko Mts., Klepa (Wagner and Horvat 1993) (170); Central Greece, Etolia, Agrinio, Agia Soufia (Wagner and Horvat 1993) (191); Central Greece, Etolia, Agrinio, Panaitoliko Mts., 3 km N of Hani Lioliou (Wagner and Horvat 1993) (202); Central Greece, Etolia, Nafpaktos, Koutsopanneika (Wagner and Horvat 1993) (203); Peloponnese, 2 km N of Peristera (Wagner and Horvat 1993) (216); Peloponnese, Aroania Mts., Kalivia (Wagner and Horvat 1993) (223); Peloponnese, Panachaiko Mts., Kounaveika (near village) (Wagner and Horvat 1993) (236); Peloponnese, Panachaiko Mts., Kristalovrisi (stream) (Wagner and Horvat 1993) (248); Peloponnese, Erymanthos Mts., Kalamata (Wagner and Horvat 1993) (249).

**Figures 9–12.**
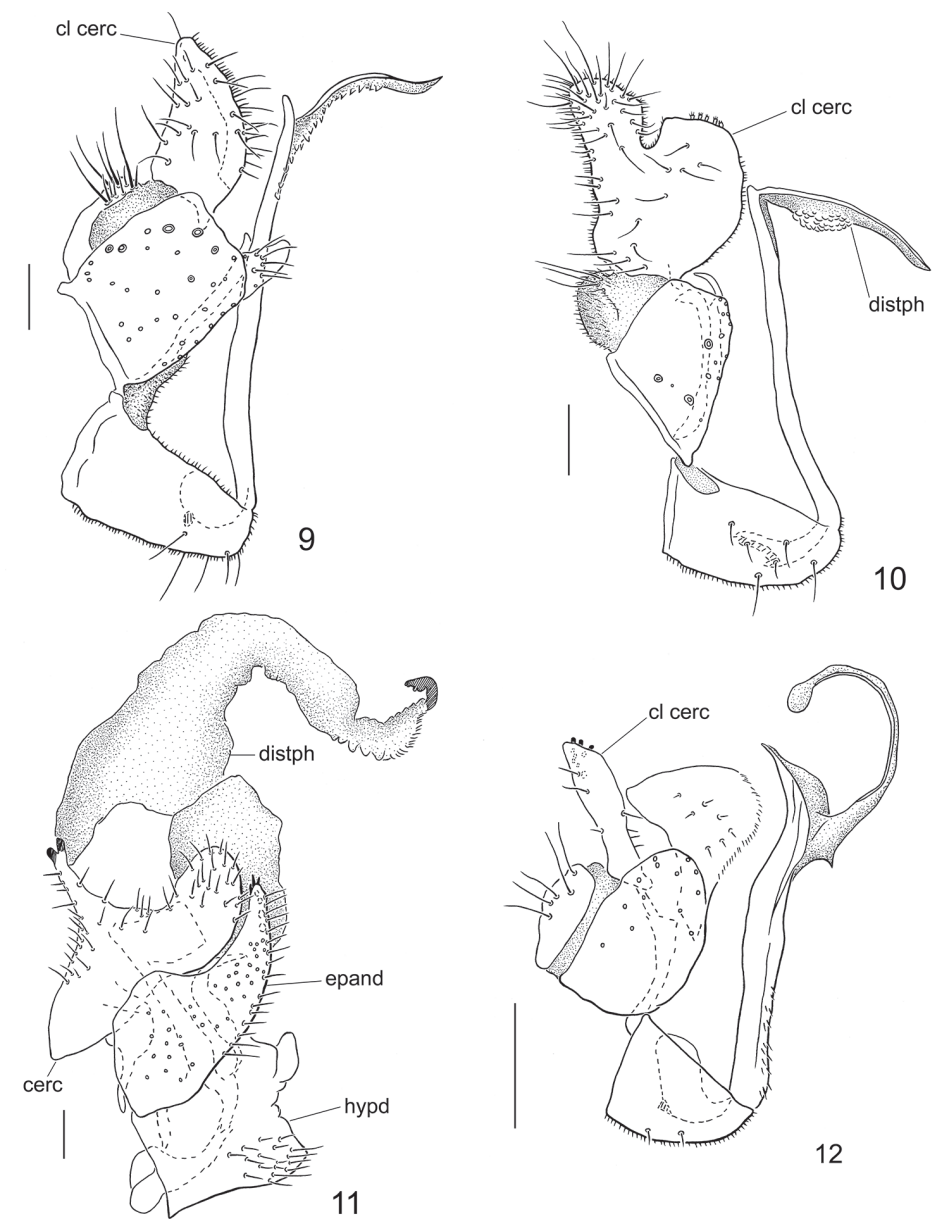
Male terminalia of *Wiedemannia* spp., lateral view **9** *W. nebulosa* Ivković & Sinclair, sp. n., paratype **10** *W. pseudoberthelemyi* Ivković & Sinclair, sp. n., paratype **11** *Chelifera horvati* Ivković & Sinclair, sp. n., holotype **12** *Clinocera megalatlantica* (Vaillant).

***Dolichocephala cretica* Wagner, 1995**

**Literature references.** Crete, stream near Kotsifiana (Wagner 1995) (257).

***Dolichocephala guttata* (Haliday, 1833)**

**Literature references.** Crete, E of Ierepetra (Wagner 1981) (251).

**New records.** Thrace, Sapka Mts. 1 (6); Thrace, Dit. Rodopi, N of Dipotama 3 (26); Macedonia, Pieria Mts., 1 (51); Macedonia, Pieria Mts. 3 (53); Epirus, N of Katarapass, 1 km SW Milea (95); Epirus, Metsovo, Katara Pass (96); Cyclades islands, Andros (206); Peloponnese, 2 km N of Peristera (216); Peloponnese, Aroania Mts., 4 km S of Solos (218); Peloponnese, Ano Potames, Kalivitis (219).

***Dolichocephala ocellata* (Costa, 1854)**

**Literature references.** North Aegean islands, Lesbos, 3 km NW of Agiasos (Wagner 1981) (130); North Aegean islands, Icaria (Wagner 1981) (140); Crete, E of Ierepetra (Wagner 1981) (251).

**New records.** Epirus, 10 km N of Louros (110); Epirus, R. Aheron, N of Gliki (115); Central Greece, Etolia, Agrinio, Agia Soufia (191).

***Dolichocephala vaillanti* Wagner, 1995**

**Literature references.** Crete, stream near Sises (Wagner 1995) (252).

***Dolichocephala zwicki* Wagner, 1995**

**Literature references.** North Aegean islands, Lesbos, 3 km NW of Agiasos (Wagner 1995) (130).

**New records.** Cyclades islands, Andros (206).

***Kowarzia barbatula* (Mik, 1880)**

**Literature references.** Thrace (Wagner 1981) (36); Macedonia, Xanthi, NE Pass Str. Xanthi-Stavroupolis (Wagner 1995) (69); North Aegean islands, Lesbos, 7 km E of Plomari (Wagner 1981) (127); North Aegean islands, Lesbos, 4 km W of Agiasos (Wagner 1981) (131); North Aegean islands, Icaria, W of Chrisostomos (Wagner 1981) (136); North Aegean islands, Chios, 2 km N of Fita (Wagner 1981) (137); North Aegean islands, Chios, N of Keramos (Wagner 1981) (138); North Aegean islands, Chios, 5 km N of Pirama (Wagner 1981) (139); North Aegean islands, Icaria (Wagner 1981) (140); Central Greece, Euboea, S of Komiton (Wagner 1995) (142).

**New records.** Thrace, W of Mega Derio (2); Thrace, Lesitse Mts. (3); Thrace, Sapka Mts., 1 (6); Thrace, 3 km N of Alexandroupoli (7); Thrace, Sapka Mts. 2 (8); Thrace, Sapka Mts., Nea Sanda 2 (10); Thrace, Anatoliki Rodopi, E od Drimi (11); Thrace, Dit. Rodopi, N of Dipotama 1 (21); Thrace, N of Dipotama 4 (23); Thrace, N of Dipotama 5 (24); Macedonia, Pieria Mts., 2 streams on Ritini (42); Macedonia, N of Agios Dimitrios (43); Macedonia, Pieria Mts., S of Elatohori (44); Macedonia, Pieria Mts., E of Fteri (46); Macedonia, Pieria Mts., 1 (51); Macedonia, Pieria Mts., 2 (52); Macedonia, Phalacro Mts., N of Livadero (55); Epirus, 10 km N of Louros (110); Epirus, R. Aheron, N of Gliki (115); Central Greece, Etolia, Vardousia Mts., Ano Chora (169); Central Greece, Etolia, Panaitoliko Mts., Klepa (170); Central Greece, Etolia, Nafpaktos, Simos (180); Central Greece, Etolia, Panaitoliko Mts., Prousos (186); Central Greece, Etolia, Panaitoliko Mts., Chaliki, Nerosirtis (188); Peloponnese, Aroania Mts., Kalivia (223); Peloponnese, Erymanthos Mts., S of Spartia (243).

***Kowarzia bipunctata* (Haliday, 1833)**

**Literature references.** North Aegean islands, Lesbos, 2 km N of Akrassi (Wagner 1981) (132); North Aegean islands, Lesbos, Ambeliko (Wagner 1981) (134); North Aegean islands, Lesbos, E of Lepetimnos (Wagner 1981) (135); Crete, E of Agios Ioannis (Wagner 1995) (250); Crete, Passas valley near Pass (Wagner 1995) (253); Crete, S of Retimnon (Wagner 1981) (254).

**New records.** Thrace, 3 km N of Alexandroupoli (7); Thrace, Sapka Mts., Nea Sanda, 2 (10); Epirus, 10 km N of Louros (110); Epirus, R. Aheron, N of Gliki (115); Central Greece, Etolia, Agrinio, Agia Soufia (191); Central Greece, Etolia, Arta, Loutraki (197); Peloponnese, village Akrata (210); Peloponnese, Aroania Mts., Kalivia (223); Peloponnese, Erymanthos Mts., Stavrohori, Eliniko (241); Peloponnese, Abelokipi (246).

***Kowarzia madicola* (Vaillant, 1965)**

**New records.** Peloponnese, Erymanthos Mts., Stavrohori, Eliniko (241).

***Kowarzia plectrum* (Mik, 1880)**

**New records.** Macedonia, Pieria Mts., E of Velventos (50); Macedonia, Pieria Mts., 1 (51); Macedonia, Pieria Mts., 2 (52); Epirus, 10 km N of Louros (110).

**Remarks.** This species is newly recorded from Greece.

***Phaeobalia dimidiata* (Loew, 1869)**

**New records.** Thrace, N of Dipotama 3 (22); Thrace, N of Dipotama 4 (23); Thrace, Dit. Rodopi, N of Dipotama 2 (25); Macedonia, Pieria Mts., E of Fteri (46); Macedonia, Pieria Mts., Fteri (47); Macedonia, Pieria Mts., W of Fteri (48); Macedonia, Pieria Mts., E of Velventos (50); Macedonia, Pieria Mts., 2 (52).

**Remarks.** This species is newly recorded from Greece.

***Roederiodes malickyi* Wagner, 1981**

**Literature references.** Crete, Xyloskalon (Wagner 1981) (256).

**Wiedemannia (Chamaedipsia) aequilobata Mandaron, 1964**

**New records.** Epirus, Lakmos Mts., 10 km S of Anilio (101).

**Wiedemannia (Chamaedipsia) ariadne Wagner, 1981**

**Literature references.** Cyclades islands, Naxos, S of Koronis (Wagner 1981) (207); Cyclades islands, Andros, Apikia (Wagner 1981) (208).

**Wiedemannia (Chamaedipsia) beckeri (Mik, 1889)**

**New records.** Thrace, Rodopi, N of Dipotama 1 (21); Thrace, N of Dipotama 3 (22); Thrace, Rodopi, N of Dipotama 3 (26); Thrace, N of Sidironero 1 (31).

**Remarks.** This species is newly recorded from Greece.

**Wiedemannia (Chamaedipsia) lota Walker, 1851**

**Literature references.** Macedonia, Olympus Mts. above Agios Dyonysos, Prionia (Wagner 1981) (41); Dodecanese islands, Rhodes, 3 km E of Archipolis (Wagner 1981) (204).

**New records.** Thrace, Anatoliki Rodopi, Drimi (12); Thrace, Anatoliki Rodopi, E of Gratini 1 (13); Thrace, Anatoliki Rodopi, E of Gratini 2 (14); Thrace, 8 km N of Sminthi (17); Thrace, N of Xanthi (18); Thrace, N of Dipotama 1 (19); Thrace, N of Dipotama 3 (22); Macedonia, N of Stavros (39); Macedonia, R. Mavroneri, 10 km W of Katerini (40); Macedonia, S of Agios Dimitrios (45); Macedonia, Pieria Mts., E of Fteri (46); Macedonia, Pieria Mts., 2 (52); Macedonia, E of Velventos (54); Macedonia, Kastoria, Nestorio (62); Macedonia, Kastoria, Grammos Mts., 7 km S Chrisi (64); Thessaly, S of Kallithea (73); Thessaly, Pieria Mts., S of Livadi (74); Thessaly, Deskati (76); Thessaly, Trikala, Longiai (77); Thessaly, S of Asprokklisia (78); Epirus, Ioannina, R. Zagoritikos, Karies (106); Epirus, Konitsa, Asimohori (109); Epirus, 10 km N of Louros (110); Epirus, S of Seriziana (111); Epirus, W of Kriopigi (114); Epirus, R. Aheron, N of Gliki (115); Epirus, Mirsini (117); Epirus, R. Kokitos, W of Gardiki (119); Epirus, Igoumenitsa, R. Thiamis, Soulopoulo (122); Epirus, Ioannina, Balndouma (124); Central Greece, Etolia, Lamia, Ieraklia (145); Central Greece, Etolia, Vardousia Mts., Paleovraha (151); Central Greece, Etolia, Nafpaktos, 9 km S of Krokilio (152); Central Greece, Etolia, Vardousia Mts., 5 km N of Grammeni Oxia (153); Central Greece, Etolia, Vardousia Mts., R. Evinos, Grammeni Oxia (154); Central Greece, Etolia, Vardousia Mts., Terpsithea (158); Central Greece, Etolia, Nafpaktos, R. Mornos, Limnitsa (159); Central Greece, Etolia, Vardousia Mts., 6 km S of Lefkada (162); Central Greece, Etolia, Vardousia Mts., 13 km S of Gardiki (164); Central Greece, Etolia, Vardousia Mts., Pougkakia (165); Central Greece, Etolia, Vardousia Mts., 2 km W of Gardiki (166); Central Greece, Etolia, Panaitoliko Mts., R. Evinos, Klepa (171); Central Greece, Etolia, R. Mornos, Nafpaktos (177); Central Greece, Etolia, Agrinio, Panaitoliko Mts., R. Evinos, Agios Dimitros (178); Central Greece, Etolia, Agrinio, Peristra, 1 km S of Perkos (182); Central Greece, Etolia, Agrinio, Panaitoliko Mts. R. Trikeriotis, Dermatio (185); Central Greece, Etolia, Agrinio, Panaitoliko Mts., Potamoula (190); Central Greece, Etolia, Lamia, Pavliani (192); Central Greece, Etolia, Agrinio, Ahlavokastro (196); Central Greece, Etolia, Arta, Loutraki (197); Central Greece, Etolia, Nafpaktos, Koutsopanneika (203); Peloponnese, R. Krathis, Voutsimos (211); Peloponnese, 3 km N of Agia Varvara (213); Peloponnese, R. Krathis, 7 km N of Peristera (214); Peloponnese, tributary of R. Krathis, 7 km N of Peristera (215); Peloponnese, R. Krathis, Peristera (217); Peloponnese, Aroania Mts., Kalivia (223); Peloponnese, Pagrati (225); Peloponnese, Aroania Mts., Kastria (226); Peloponnese, Kato Klitoria (227); Peloponnese, Aroania Mts., Xelmos (above) (228); Peloponnese, R. Piro, Elliniko (230); Peloponnese, Panachaiko Mts., tributory of R. Selinous, Leontio (231); Peloponnese, Panachaiko Mts., Leontio (232); Peloponnese, Erymanthos Mts., Lechouri (233); Peloponnese, Panachaiko Mts., Veteika (234); Peloponnese, Erymanthos Mts., Kato Vlasia (235); Peloponnese, Panachaiko Mts., Kounaveika (near village) (236); Peloponnese, Erymanthos Mts., Profitis Ilias (239); Peloponnese, Erymanthos Mts., Stavrohori, Eliniko (241); Peloponnese, Panachaiko Mts., Souli (242); Peloponnese, Erymanthos Mts., Manesi (244); Peloponnese, E of Olympia (247).

**Wiedemannia (Eucelidia) zetterstedti (Fallén, 1826)**

**Literature references.** Thrace (Wagner 1981) (36); Macedonia, Olympus Mts., above Agios Dyonysos, Prionia (Wagner 1981) (41); Epirus, Preveza, Zalongu, stream 2 km E of Mirsini (Wagner 1995) (113); North Aegean islands, Samos, below Manolates (Wagner 1981) (125); North Aegean islands, Samos, E of Pirgos (Wagner 1981) (126); North Aegean islands, Lesbos, 1 km SW of Megalochori (Wagner 1981) (129); North Aegean islands, Lesbos, S of Neochorion (Wagner 1981) (133); Central Greece, Euboea, S of Komiton (Wagner 1995) (142); Central Greece, Euboea, Steni Dirfyos (former Ano Steni) (Wagner 1995) (143); Central Greece, Parnassus Mts., above Polydrosos (Wagner 1981) (146); Central Greece, Central Euboea (Wagner 1981) (201); Cyclades islands, Andros, Apikia (Wagner 1981) (208); Peloponnese, Taygetos Mts. (below the summit) (Wagner 1981) (209); Laschtabend (Alpen) (Wagner 1981) (258).

**New records.** Thrace, E of Mega Derio (1); Thrace, N of Avas (5); Thrace, Sapka Mts. 1 (6); Thrace, Sapka Mts. 2 (8); Thrace, Sapka Mts., Nea Sanda 1 (9); Thrace, Sapka Mts., Nea Sanda 2 (10); Thrace, Anatoliki Rodopi, E od Drimi (11); Thrace, Anatoliki Rodopi, Drimi (12); Thrace, Anatoliki Rodopi, E of Gratini 1 (13); Thrace, Miki (16); Thrace, 8 km N of Sminthi (17); Thrace, N of Xanthi (18); Thrace, N of Dipotama 1 (19); Thrace, N of Dipotama 2 (20); Thrace, Dit. Rodopi, N of Dipotama 1 (21); Thrace, N of Dipotama 3 (22); Thrace, N of Dipotama 5 (24); Thrace, S of Dipotama (27); Thrace, S of Silli (28); Thrace, Dit. Rodopi, Skaloti (29); Thrace, W of Sidironero (34); Thrace, Rodopi, E of Mikromilia (35); Macedonia, E of Mikroklisoura (38); Macedonia, N of Stavros (39); Macedonia, R. Mavroneri, 10 km W of Katerini (40); Macedonia, Pieria Mts., 2 streams on Ritini (42); Macedonia, N of Agios Dimitrios (43); Macedonia, S of Agios Dimitrios (45); Macedonia, Pieria Mts., E of Fteri (46); Macedonia, Pieria Mts., Fteri (47); Macedonia, Pieria Mts., W of Fteri (48); Macedonia, W of Daskio (49); Macedonia, Pieria Mts., 1 (51); Macedonia, Pieria Mts., 2 (52); Macedonia, Pieria Mts., 3 (53); Macedonia, Phalacro Mts., N of Livadero (55); Macedonia, Grevena, 6 km S of Milea (57); Macedonia, Kozani, Polilako (Paraveti), Neapolis (59); Macedonia, Smokilas Mts., main stream near the bridge, 2 km E of Agia Paraskevi (63); Thessaly, Ossa Mts., stream Apataniana (71); Thessaly, S of Kallithea (73); Thessaly, Deskati (76); Thessaly, S of Asprokklisia (78); Thessaly, Kalambaka, Agios Nikolaos (80); Thessaly, Trikala, Kato Palagokaria (82); Thessaly, Kalambaka, 5 km E of Paleochori (83); Thessaly, Kalambaka, Trigona (85); Thessaly, Kalambaka, Koridallos (86); Thessaly, Trikala, Arta, Pahtouri (87); Thessaly, Kalambaka, 4 km S of Ambelia (91); Epirus, Metsovo, 14 km S of Milea (92); Epirus, Metsovo, R. Metsovitikos (99); Epirus, Metsovo, Lakmos Mts., Anthohori, (bellow rapid river) (100); Epirus, Ioannina, R. Zagoritikos, Karies (106); Epirus, 10 km N of Louros (110); Epirus, S of Seriziana (111); Epirus, Ioannina, R. Voidomatis, Aristi (112); Epirus, R. Aheron, N of Gliki (115); Epirus, Kanallaki, Skepaston (116); Epirus, Mirsini (117); Epirus, R. Aheron, Gliki (118); Epirus, R. Kokitos, W of Gardiki (119); Epirus, Igoumenitsa, R. Thiamis, Soulopoulo (122); Central Greece, Etolia, Lamia, Ieraklia (145); Central Greece, Etolia, Vardousia Mts., Mousonitsa (149); Central Greece, Etolia, Vardousia Mts., Athanasios Diakos (150); Central Greece, Etolia, Vardousia Mts., Paleovraha (151); Central Greece, Etolia, Nafpaktos, 9 km S of Krokilio (152); Central Greece, Etolia, Vardousia Mts., 7 km N of Grammeni Oxia (156); Central Greece, Etolia, Nafpaktos, R. Mornos, Limnitsa (159); Central Greece, Etolia, Vardousia Mts., Elato (161); Central Greece, Etolia, Vardousia Mts., Pougkakia (165); Central Greece, Etolia, Vardousia Mts., 2 km W of Gardiki (166); Central Greece, Etolia, Vardousia Mts., Grigorio (167); Central Greece, Etolia, Vardousia Mts., Ano Chora (169); Central Greece, Etolia, Panaitoliko Mts., Klepa (170); Central Greece, Etolia, Panaitoliko Mts., R. Evinos, Klepa (171); Central Greece, Etolia, Vardousia Mts., 3 km W of Kryoneri (172); Central Greece, Etolia, Vardousia Mts., Kato Chora (173); Central Greece, Etolia, Nafpaktos, Anthofito (174); Central Greece, Etolia, Nafpaktos, tributory of R. Evinos, 6 km N of Pokista (176); Central Greece, Etolia, R. Mornos, Nafpaktos (177); Central Greece, Etolia, Nafpaktos, Simos (180); Central Greece, Etolia, Nafpaktos, Pokista (181); Central Greece, Etolia, Agrinio, Peristra, 1 km S of Perkos (182); Central Greece, Etolia, Agrinio, R. Evinos, Kato Hrisovitsa, Diasellaki (183); Central Greece, Etolia, Panaitoliko Mts., Chaliki, Ladikon (187); Central Greece, Etolia, Agrinio, Agia Soufia (191); Central Greece, Etolia, Lamia, Pavliani (192); Central Greece, Etolia, Giona Mts., Sikia (194); Central Greece, Oeta Mts., stream Valorema, Pavliani (195); Central Greece, Etolia, Agrinio, Ahlavokastro (196); Central Greece, Etolia, Arta, Loutraki (197); Central Greece, Etolia, Agrinio, Panaitoliko Mts., Palagohori (199); Central Greece, Etolia, Nafpaktos, Avrorema bridge (200); Central Greece, Etolia, Agrinio, Panaitoliko Mts., 3 km N of Hani Lioliou (202); Central Greece, Etolia, Nafpaktos, Koutsopanneika (203); Peloponnese, Aroania Mts., 2 km S of Zarouchla (212); Peloponnese, Aroania Mts., Zarouhla (220); Peloponnese, Aroania Mts., Kalivia (223); Peloponnese, Kato Klitoria (227); Peloponnese, Panachaiko Mts., tributory of R. Selinous, Leontio (231); Peloponnese, Erymanthos Mts., Lechouri (233); Peloponnese, Panachaiko Mts., Veteika (234); Peloponnese, Panachaiko Mts., Kounaveika (near village) (236); Peloponnese, E of Olympia (247).

**Wiedemannia (Philolutra) angelieri Vaillant, 1967**

**New records.** Central Greece, Etolia, Vardousia Mts., Athanasios Diakos (150).

**Remarks.** This species is newly recorded from Greece.

**Wiedemannia (Philolutra) chvali Joost, 1981**

**New records.** Thrace, N of Dipotama 3 (22); Thrace, N of Sidironero 1 (31).

**Remarks.** This species is newly recorded from Greece.

**Wiedemannia (Philolutra) fallaciosa (Loew, 1873)**

**Literature references.** Macedonia, Olympus Mts. above Agios Dyonysos, Prionia (Wagner 1981) (41); Epirus, Preveza, Zalongu, stream 2 km E of Mirsini (Wagner 1995) (113).

**New records.** Thrace, E of Mega Derio (1); Thrace, Miki (16); Thrace, N of Dipotama 1 (19); Thrace, Dit. Rodopi, Skaloti (29); Macedonia, R. Mavroneri, 10 km W of Katerini (40); Macedonia, Pieria Mts., S of Elatohori (44); Macedonia, S of Agios Dimitrios (45); Macedonia, Pieria Mts., E of Fteri (46); Macedonia, W of Daskio (49); Macedonia, Pieria Mts., 2 (52); Macedonia, E of Velventos (54); Macedonia, Grevena, Milea (56); Macedonia, Grevena, 6 km S of Milea (57); Macedonia, Kozani, Polilako (Paraveti), Neapolis (59); Macedonia, Grevena, R. Venetikos, Kipourio (60); Macedonia, Kastoria, Nestorio (62); Macedonia, Kastoria, Grammos Mts., 7 km S Chrisi (64); Macedonia, Kastoria, Grammos Mts., 6 km N Pefkofito (65); Thessaly, Deskati (76); Thessaly, S of Asprokklisia (78); Thessaly, Trikala, Moshofito, Avra (79); Thessaly, Kalambaka, Agios Nikolaos (80); Thessaly, Trikala, Stournareika (81); Thessaly, Trikala, Kato Palagokaria (82); Thessaly, Kalambaka, 5 km E of Paleochori (83); Thessaly, Kalambaka, Paleochori (84); Thessaly, Kalambaka, Trigona (85); Thessaly, Trikala, Arta, Pahtouri (87); Thessaly, Trikala, Arta, R. Ahelos, Kapsala (88); Thessaly, Trikala, Arta, Korifi (89); Epirus, Metsovo, 14 km S of Milea (92); Epirus, Metsovo, Lakmos Mts., Anilio (5 km S bellow river) (93); Epirus, Metsovo, 12 km W Milea (98); Epirus, Metsovo, R. Metsovitikos (99); Epirus, Metsovo, Lakmos Mts., Anthohori, (bellow rapid river) (100); Epirus, Lakmos Mts., 10 km S of Anilio (101); Epirus, Ioannina, Megalo Peristeri (104); Epirus, Ioannina, R. Zagoritikos, Karies (106); Epirus, Konitsa, Asimohori (109); Epirus, 10 km N of Louros (110); Epirus, Ioannina, R. Voidomatis, Aristi (112); Epirus, W of Kriopigi (114); Epirus, R. Aheron, N of Gliki (115); Epirus, Kanallaki, Skepaston (116); Epirus, Mirsini (117); Epirus, R. Kokitos, W of Gardiki (119); Epirus, Ioannina, Balndouma (124); Central Greece, Etolia, Lamia, Ieraklia (145); Central Greece, Etolia, Vardousia Mts., Stromi (148); Central Greece, Etolia, Vardousia Mts., Mousonitsa (149); Central Greece, Etolia, Vardousia Mts., Athanasios Diakos (150); Central Greece, Etolia, Nafpaktos, 9 km S of Krokilio (152); Central Greece, Etolia, Vardousia Mts., 5 km N of Grammeni Oxia (153); Central Greece, Etolia, Vardousia Mts., R. Evinos, Grammeni Oxia (154); Central Greece, Etolia, Vardousia Mts., 9 km N of Grammeni Oxia (155); Central Greece, Etolia, Vardousia Mts., 7 km N of Grammeni Oxia (156); Central Greece, Etolia, Vardousia Mts., Terpsithea (158); Central Greece, Etolia, Nafpaktos, R. Mornos, Limnitsa (159); Central Greece, Etolia, Vardousia Mts., Elatovrisi (160); Central Greece, Etolia, Vardousia Mts., Elato (161); Central Greece, Etolia, Vardousia Mts., 6 km S of Lefkada (162); Central Greece, Etolia, Vardousia Mts., Gardiki (163); Central Greece, Etolia, Vardousia Mts., 13 km S of Gardiki (164); Central Greece, Etolia, Vardousia Mts., Pougkakia (165); Central Greece, Etolia, Vardousia Mts., 2 km W of Gardiki (166); Central Greece, Etolia, Vardousia Mts., Grigorio (167); Central Greece, Etolia, Panaitoliko Mts., Klepa (170); Central Greece, Etolia, Panaitoliko Mts., R. Evinos, Klepa (171); Central Greece, Etolia, Vardousia Mts., 3 km W of Kryoneri (172); Central Greece, Etolia, Vardousia Mts., Kato Chora (173); Central Greece, Etolia, Nafpaktos, Anthofito (174); Central Greece, Etolia, Nafpaktos, tributory of R. Evinos, 6 km N of Pokista (176); Central Greece, Etolia, R. Mornos, Nafpaktos (177); Central Greece, Etolia, Agrinio, Panaitoliko Mts., R. Evinos, Agios Dimitros (178); Central Greece, Etolia, Nafpaktos, 2 km N of Pokista (179); Central Greece, Etolia, Nafpaktos, Simos (180); Central Greece, Etolia, Nafpaktos, Pokista (181); Central Greece, Etolia, Agrinio, Peristra, 1 km S of Perkos (182); Central Greece, Etolia, Agrinio, R. Evinos, Kato Hrisovitsa, Diasellaki (183); Central Greece, Etolia, Agrinio, Panaitoliko Mts. R. Trikeriotis, Dermatio (185); Central Greece, Etolia, Lamia, Pavliani (192); Central Greece, Etolia, Giona Mts., Sikia (194); Central Greece, Etolia, Agrinio, Ahlavokastro (196); Central Greece, Etolia, Arta, Loutraki (197); Central Greece, Etolia, Agrinio, Panaitoliko Mts., Palagohori (199); Central Greece, Etolia, Nafpaktos, Avrorema bridge (200); Central Greece, Etolia, Agrinio, Panaitoliko Mts., 3 km N of Hani Lioliou (202); Central Greece, Etolia, Nafpaktos, Koutsopanneika (203); Peloponnese, R. Krathis, Voutsimos (211); Peloponnese, 3 km N of Agia Varvara (213); Peloponnese, R. Krathis, 7 km N of Peristera (214); Peloponnese, tributary of R. Krathis, 7 km N of Peristera (215); Peloponnese, 2 km N of Peristera (216); Peloponnese, R. Krathis, Peristera (217); Peloponnese, Ano Potames, Kalivitis (219); Peloponnese, Aroania Mts., Zarouhla (220); Peloponnese, Likouria (under the village) (222); Peloponnese, Aroania Mts., Kalivia (223); Peloponnese, Aroania Mts., Kastria (226); Peloponnese, Kato Klitoria (227); Peloponnese, Aroania Mts., Xelmos (above) (228); Peloponnese, R. Piro, Elliniko (230); Peloponnese, Panachaiko Mts., tributory of R. Selinous, Leontio (231); Peloponnese, Panachaiko Mts., Leontio (232); Peloponnese, Erymanthos Mts., Lechouri (233); Peloponnese, Panachaiko Mts., Veteika (234); Peloponnese, Erymanthos Mts., Kato Vlasia (235); Peloponnese, Panachaiko Mts., Kounaveika (near village) (236); Peloponnese, Panachaiko Mts., Moira (237); Peloponnese, Panachaiko Mts., Moira (after village) (238); Peloponnese, Panachaiko Mts., Souli (242); Peloponnese, Abelokipi (246); Peloponnese, E of Olympia (247).

**Wiedemannia (Pseudowiedemannia) lamellata (Loew, 1869)**

**Literature references.** Thessaly, Karya (Wagner 1981) (72); North Aegean islands, Lesbos, 1 km W of Ippion (Wagner 1981) (128).

**New records.** Thrace, Sapka Mts., Nea Sanda 1 (9); Thrace, Anatoliki Rodopi, E od Drimi (11); Thrace, Anatoliki Rodopi, Drimi (12); Thrace, Anatoliki Rodopi, E of Gratini 1 (13); Thrace, 8 km N of Sminthi (17); Thrace, S of Silli (28); Thrace, Dit. Rodopi, Skaloti (29); Thrace, Dit. Rodopi 1 (30); Thrace, N of Sidironero 1 (31); Thrace, Dit. Rodopi 2 (32); Thrace, N of Sidironero 2 (33); Thrace, W of Sidironero (34); Macedonia, N of Stavros (39); Macedonia, R. Mavroneri, 10 km W of Katerini (40); Macedonia, Phalacro Mts., N of Livadero (55); Macedonia, Kozani, Polilako (Paraveti), Neapolis (59); Thessaly, Trikala, Kato Palagokaria (82); Thessaly, Kalambaka, 5 km E of Paleochori (83); Thessaly, Kalambaka, Paleochori (84); Thessaly, Kalambaka, Koridallos (86); Epirus, Metsovo, Lakmos Mts., Anthohori, (bellow rapid river) (100); Epirus, Ioannina, R. Vardas, Abelos (123); Central Greece, Etolia, Lamia, Ieraklia (145); Central Greece, Etolia, Vardousia Mts., 7 km N of Grammeni Oxia (156); Central Greece, Etolia, Vardousia Mts., 7 km S of Gardiki (157); Central Greece, Etolia, Vardousia Mts., Terpsithea (158); Central Greece, Etolia, Vardousia Mts., 13 km S of Gardiki (164); Central Greece, Etolia, Vardousia Mts., Pougkakia (165); Central Greece, Etolia, Vardousia Mts., 2 km W of Gardiki (166); Central Greece, Etolia, Agrinio, Panaitoliko Mts., Megali Chora (193); Central Greece, Etolia, Nafpaktos, Avrorema bridge (200); Peloponnese, Aroania Mts., Kalivia (223); Peloponnese, Aroania Mts., Kastria (226); Peloponnese, Panachaiko Mts., tributory of R. Selinous, Leontio (231); Peloponnese, Panachaiko Mts., Leontio (232); Peloponnese, Erymanthos Mts., Lechouri (233); Peloponnese, Panachaiko Mts., Veteika (234); Peloponnese, Erymanthos Mts., Kato Vlasia (235); Peloponnese, Panachaiko Mts., Kounaveika (near village) (236); Peloponnese, Erymanthos Mts., Manesi (244); Peloponnese, E of Olympia (247).

**Wiedemannia (Pseudowiedemannia) microstigma (Bezzi, 1904)**

**New records.** Thessaly, Trikala, Kato Palagokaria (82); Central Greece, Etolia, Vardousia Mts., Stromi (148).

**Wiedemannia (Roederella) czernyi (Bezzi, 1905)**

**Literature references.** Macedonia, Chalkidiki, Chlomon Oros., Paleokastron, Vatonia P. 1 (Wagner 1995) (66).

**New records.** Thrace, E of Sapka Mts., big stream in the valley (4); Macedonia, Chalkidiki, Chlomon Oros., Paleokastron, Vatonia P. 2 (67).

**Wiedemannia (Wiedemannia) andreevi Joost, 1982**

**New records.** Thrace, S of Silli (28).

**Wiedemannia (Wiedemannia) bilobata Oldenberg, 1910**

**Literature references.** Macedonia, Olympus Mts. above Agios Dyonysos, Prionia (Wagner 1981) (42); Central Greece, Parnassus Mts., above Polydrosos (Wagner 1981) (146).

**Wiedemannia (Wiedemannia) dinarica Engel, 1940**

**New records.** Epirus, Ioannina, R. Voidomatis, Aristi (112); Epirus, R. Aheron, N of Gliki (115); Epirus, R. Aheron, Gliki (118); Peloponnese, Likouria (under the village) (222); Peloponnese, Aroania Mts., Krinofita (224); Peloponnese, Aroania Mts., Kastria (226); Peloponnese, Kato Klitoria (227).

**Wiedemannia (Wiedemannia) dyonysica Wagner, 1990**

**Literature references.** Macedonia, Olympus Mts. above Agios Dyonysos, Prionia (Wagner 1990) (41).

**Wiedemannia (Wiedemannia) graeca Vaillant & Wagner, 1990**

**Literature references.** Central Greece, Polydrosos (Vaillant and Wagner 1990) (144).

**New records.** Thrace, Rodopi, Skaloti (29); Thessaly, Trikala, Stournareika (81); Thessaly, Kalambaka, 5 km E of Paleochori (83); Thessaly, Kalambaka, Paleochori (84); Thessaly, Trikala, Arta, R. Ahelos, Kapsala (88); Epirus, Metsovo, Lakmos Mts., 2 km S of Anilio (bellow left tributary) (97); Epirus, Metsovo, Lakmos Mts., Anthohori, (bellow rapid river) (100); Central Greece, Etolia, Vardousia Mts., Stromi (148).

**Wiedemannia (Wiedemannia) tricuspidata (Bezzi, 1905)**

**New records.** Thrace, S of Silli (28); Macedonia, Grevena, R. Venetikos, Kipourio (60); Macedonia, Kastoria, Grammos Mts., 7 km S Chrisi (64); Thessaly, Trikala, Longiai (77); Thessaly, Trikala, Kato Palagokaria (82); Epirus, Konitsa, R. Saradaporos, Drosopigi (108); Central Greece, Etolia, Nafpaktos, R. Mornos, Limnitsa (159); Central Greece, Etolia, Panaitoliko Mts., R. Evinos, Klepa (171); Central Greece, Etolia, Nafpaktos, tributory of R. Evinos, 6 km N of Pokista (176); Central Greece, Etolia, R. Mornos, Nafpaktos (177); Central Greece, Etolia, Agrinio, Panaitoliko Mts., R. Evinos, Agios Dimitros (178); Central Greece, Etolia, Agrinio, Peristra, 1 km S of Perkos (182).

***Wiedemania artemisa* Ivković & Plant, 2012**

**Literature references.** Thessaly, Trikala, Kato Palagokaria (Ivković et al. 2012) (82); Thessaly, Trikala, Arta, Pahtouri (Ivković et al. 2012) (87); Thessaly, Trikala, Arta, R. Ahelos, Kapsala (Ivković et al. 2012) (88); Thessaly, Trikala, Arta, Korifi (Ivković et al. 2012) (89); Epirus, Metsovo, Lakmos Mts., Anthohori, (bellow rapid river) (Ivković et al. 2012) (100); Epirus, Igoumenitsa, R. Thiamis, Soulopoulo (Ivković et al. 2012) (122); Central Greece, Etolia, Lamia, Ieraklia (Ivković et al. 2012) (145); Central Greece, Etolia, Vardousia Mts., 7 km S of Gardiki (Ivković et al. 2012) (157); Central Greece, Etolia, Vardousia Mts., Pougkakia (Ivković et al. 2012) (165); Peloponnese, R. Krathis, Voutsimos (Ivković et al. 2012) (211); Peloponnese, R. Krathis, Peristera (Ivković et al. 2012) (217); Peloponnese, Likouria (under the village) (Ivković et al. 2012) (222); Peloponnese, Aroania Mts., Kastria (Ivković et al. 2012) (226); Peloponnese, Kato Klitoria (Ivković et al. 2012) (227); Peloponnese, Panachaiko Mts., tributory of R. Selinous, Leontio (Ivković et al. 2012) (231); Peloponnese, Panachaiko Mts., Leontio (Ivković et al. 2012) (232); Peloponnese, Panachaiko Mts., Veteika (Ivković et al. 2012) (234); Peloponnese, Panachaiko Mts., Souli (Ivković et al. 2012) (242).

**New records.** Thessaly, Kalambaka, 4 km S of Ambelia (91); Epirus, Metsovo, Lakmos Mts., 2 km S of Anilio (bellow left tributary) (97); Epirus, Konitsa, Smolikas Mts., Pournia (107); Epirus, Mirsini (117); Central Greece, Etolia, Vardousia Mts., Stromi (148); Central Greece, Etolia, Vardousia Mts., Athanasios Diakos (150); Central Greece, Etolia, Nafpaktos, 9 km S of Krokilio (152); Central Greece, Etolia, Vardousia Mts., R. Evinos, Grammeni Oxia (154); Central Greece, Etolia, Vardousia Mts., 7 km N of Grammeni Oxia (156); Central Greece, Etolia, Vardousia Mts., Terpsithea (158); Central Greece, Etolia, Nafpaktos, R. Mornos, Limnitsa (159); Central Greece, Etolia, Vardousia Mts., 13 km S of Gardiki (164); Central Greece, Etolia, Vardousia Mts., 2 km W of Gardiki (166); Central Greece, Etolia, Vardousia Mts., Grigorio (167); Central Greece, Etolia, Vardousia Mts., Kato Chora (173); Central Greece, Karpenisi, Agios Nikolaos (175); Central Greece, Etolia, Nafpaktos, tributory of R. Evinos, 6 km N of Pokista (176); Central Greece, Etolia, Nafpaktos, Pokista (181); Central Greece, Etolia, Agrinio, Peristra, 1 km S of Perkos (182); Central Greece, Etolia, Panaitoliko Mts., Prousos (186); Central Greece, Etolia, Panaitoliko Mts., Chaliki, Ladikon (187); Central Greece, Etolia, Agrinio, Panaitoliko Mts., Anatoliki Frangista (189); Central Greece, Etolia, Lamia, Pavliani (192); Central Greece, Etolia, Agrinio, Panaitoliko Mts., Megali Chora (193); Central Greece, Etolia, Agrinio, Panaitoliko Mts., Houni (198); Central Greece, Etolia, Nafpaktos, Koutsopanneika (203); Peloponnese, 3 km N of Agia Varvara (213); Peloponnese, R. Krathis, 7 km N of Peristera (214).

***Wiedemannia iphigeniae* Ivković & Sinclair, sp. n.**

**Records.** Peloponnese, Aroania Mts., Krinofita (224).

***Wiedemannia ljerkae* Ivković & Sinclair, sp. n.**

**Records.** Epirus, Igoumenitsa, R. Thiamis, Soulopoulo (122); Central Greece, Etolia, Agrinio, Peristra, 1 km S of Perkos (182); Peloponnese, Aroania Mts., Kastria (226); Peloponnese, Kato Klitoria (227).

***Wiedemannia nebulosa* Ivković & Sinclair, sp. n.**

**Records.** Thrace, N of Dipotama 5 (24).

***Wiedemannia pseudoberthelemyi* Ivković & Sinclair, sp. n.**

**Records.** Central Greece, Etolia, Vardousia Mts., R. Evinos, Grammeni Oxia (154); Central Greece, Etolia, R. Mornos, Nafpaktos (177); Central Greece, Panaitoliko Mts., R. Tavropos, Kalesmeno (184); Central Greece, Etolia, Agrinio, Agia Soufia (191).

### Subfamily Hemerodromiinae

***Chelifera angusta* Collin, 1927**

**New records.** North Aegean islands, Lesbos (141).

**Remarks.** This species is newly recorded from Greece.

***Chelifera barbarica* Vaillant, 1982**

**Literature references.** Dodecanese islands, Rhodes, near Archipolis (Wagner 1995) (205).

***Chelifera horvati* Ivković & Sinclair, sp. n.**

**Records.** Central Greece, Etolia, Arta, Loutraki (197).

***Chelifera precabunda* Collin, 1961**

**New records.** Thrace, Sapka Mts., 1 (6); Thrace, Dit. Rodopi, Skaloti (29); Thrace, Rodopi, E of Mikromilia (35); Macedonia, Pieria Mts., E of Velventos (50); Peloponnese, R. Krathis, 7 km N of Peristera (214).

***Chelifera precatoria* (Fallén, 1816)**

**Literature references.** Crete, Georgioupolis (Wagner 1981) (255).

***Chelifera stigmatica* (Schiner, 1862)**

**Literature references.** North Aegean islands, Samos, E of Pirgos (Wagner 1981) (126).

**New records.** Thrace, N of Sidironero 2 (33); Thessaly, Trikala, Kato Palagokaria (82); Epirus, 10 km N of Louros (110); Epirus, R. Aheron, N of Gliki (115); Central Greece, Etolia, Vardousia Mts., Stromi (148); Central Greece, Etolia, Panaitoliko Mts., R. Evinos, Klepa (171); Central Greece, Etolia, Agrinio, Peristra, 1 km S of Perkos (182); Central Greece, Etolia, Nafpaktos, Koutsopanneika (203); Peloponnese, Erymanthos Mts., Stavrohori, Eliniko (241); Peloponnese, E of Olympia (247).

***Chelifera trapezina* (Zetterstedt, 1838)**

**Literature references.** North Aegean islands, Samos, E of Pirgos (Wagner 1981) (126).

***Hemerodromia melangyna* Collin, 1927**

**New records.** Epirus, 10 km N of Louros (110); Epirus, R. Aheron, N of Gliki (115).

**Remarks.** This species is newly recorded from Greece.

***Hemerodromia oratoria* (Fallén, 1816)**

**Literature references.** Peloponnese, Ano Kastritsi, stream (Wagner 1995) (240).

**New records.** Thrace, Lesitse Mts. (3); Thrace, Anatoliki Rodopi, E od Drimi (11); Thrace, Anatoliki Rodopi, Drimi (12); Thrace, Miki (16); Thrace, 8 km N of Sminthi (17); Epirus, 10 km N of Louros (110); Epirus, W of Kriopigi (114); Epirus, Mirsini (117); Central Greece, Etolia, Lamia, Ieraklia (145).

***Hemerodromia unilineata* Zetterstedt, 1842**

**Literature references.** Thessaly, Portaria (Wagner 1995) (70).

**New records.** Thrace, Anatoliki Rodopi, E od Drimi (11); Thrace, Anatoliki Rodopi, Drimi (12); Thrace, Anatoliki Rodopi, E of Gratini, 1 (13); Thrace, 8 km N of Sminthi (17); Thrace, S of Silli (28); Thrace, W of Sidironero (34); Macedonia, E of Mikroklisoura (38); Macedonia, W of Daskio (49); Epirus, 10 km N of Louros (110); Epirus, R. Aheron, N of Gliki (115); Epirus, Mirsini (117); Epirus, R. Kokitos, Themelo (120); Epirus, Igoumenitsa, Thesprotia, R. Thiamis, Neohori (121).

## Results and discussion

**Figure 13.**
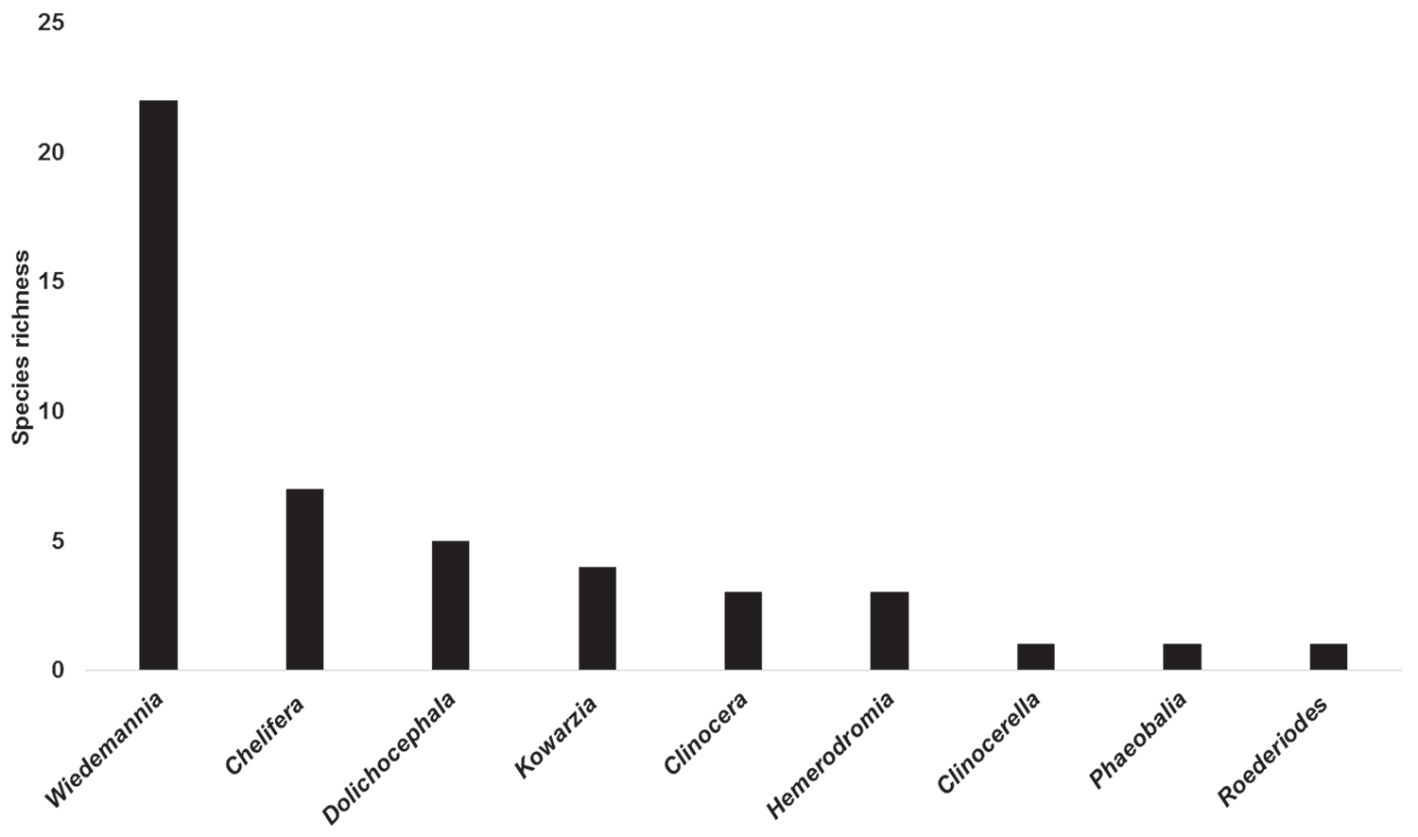
Species richness of aquatic Empididae genera from Greece.

**Species richness and assemblage composition.** A total of 47 species of aquatic empidids are recorded from Greece (Table 2), collected from 258 sites (Fig. 1, Table 1). The subfamily Clinocerinae is represented by 37 species, in seven genera: *Clinocera* Meigen (3 species), *Clinocerella* Engel (1 species), *Dolichocephala* Macquart (5 species), *Kowarzia* Mik (4 species), *Phaeobalia* (1 species), *Roederiodes* Coquillett (1 species) and *Wiedemannia* Zetterstedt (22 species). The subfamily Hemerodromiinae is represented by 10 species, in two genera: *Chelifera* (7 species) and *Hemerodromia* Meigen (3 species) (Table 2). The Clinocerinae genus *Wiedemannia* is most species rich (46.8%), followed by the Hemerodromiinae genus *Chelifera* (14.9%) (Fig. 13). The Hellenic Western Balkan (Ecoregion 6) is the richest European Ecoregion with 42 species, while 20 species are recorded from the Eastern Balkan (Ecoregion 7), and 15 species occur in both ecoregions (Table 2). Most aquatic Empididae inhabiting Greece are widely distributed in Europe or more broadly, but 10 species are only known from mainland Greece or its islands (Table 2).

**Figure 14.**
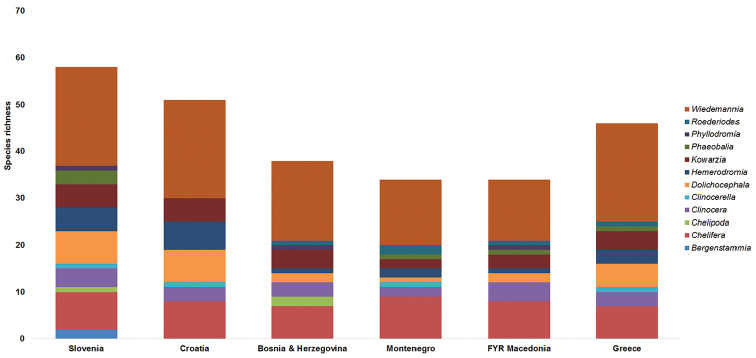
Comparison of the Greek aquatic Empididae assemblage with those of other Balkan countries.

Greece supports at least 47 species, but this is unlikely to be the final number. Slovenia, situated in the northwest part of the Balkans, supports 58 species, Croatia 51 species, while Bosnia & Herzegovina, Montenegro and FYR Macedonia have 38, 34 and 34, respectively (Fig. 14). The Sørensen Index of Similarity showed that the Empididae fauna of Greece is most similar to that of FYR Macedonia followed by Bosnia & Herzegovina, whereas it is the least similar to that of Montenegro (Table 3).

**Table 3.** Sørensen Index of Similarity between aquatic dance fly assemblages of studied Balkan countries in relation to Greece. Abbreviations: SLO = Slovenia, HR = Croatia, B&H = Bosnia & Herzegovina, MN = Montenegro, FYRM = FYR Macedonia, GR = Greece.

	**SLO**	**HR**	**B&H**	**MN**	**FYRM**	**GR**
**SLO**	0					
**HR**	71.56	0				
**B&H**	54.16	62.92	0			
**MN**	41.3	61.17	61.11	0		
**FYRM**	47.83	56.47	61.11	52.94	0	
**GR**	45.71	48.97	51.76	34.56	51.85	0

We compared our list of Greek species with existing checklists in “Fauna Europaea” (Chvála 2012) and the World Catalogue of Empididae (Yang et al. 2007). The following species were not recorded from Greece in both these works: *Chelifera angusta* and *Hemerodromia melangyna* from the subfamily Hemerodromiinae, and *Clinocera megalatlantica*, *Kowarzia plectrum*, *Phaeobalia dimidiata*, W. (Chamaedipsia) beckeri, W. (Philolutra) angelieri and W. (P.) chvali from the subfamily Clinocerinae. They represent new country records. On the other hand, some species that are listed in Chvála (2012) and Yang et al. (2007) are not included in the present checklist. We omitted Wiedemannia (Philolutra) hygrobia (Loew) because its presence has not been confirmed in Greece. However, it is possible that it does occur in Greece as it is present in surrounding countries (Chvála 2012, Horvat 1995b, 1997) and consequently it was included in the above key to species. Altogether, 13 species (including the new species) are recorded for the first time from Greece. The species richness of both subfamilies varies between European Ecoregions.

Clinocerinae show greater species richness in mountainous areas of Europe (Vaillant 1982, Wagner and Gathmann 1996), and they are also more species rich in streams and rivers in the Balkans (Horvat 1993, 1995b, 1997, Ivković et al. 2007, 2010, 2012, 2013a, 2013b, 2014).

**Comparison with neighbouring faunas.** Greece has been divided into two ecoregions: Hellenic Western Balkan (Ecoregion 6) and Eastern Balkan (Ecoregion 7). The higher species richness is in the Hellenic Western Balkan Ecoregion, but the Eastern Balkan Ecoregion in Greece is much smaller, so this was an expected result. Greece supports at least 47 species, of which 10 are currently endemic to the country (*Dolichocephala cretica*, *D. vaillanti*, *Clinocerella siveci*, *Roederiodes malickyi*, Wiedemannia (W.) graeca, *W. iphigeniae*, *W. ljerkae*, *W. nebulosa*, *W. pseudoberthelemyi*, *Chelifera horvati*). The higher number of species recorded for Slovenia and the far fewer species recorded, for instance, in Montenegro, FYR Macedonia and Bosnia & Herzegovina should be viewed with caution. Slovenia was well studied (Horvat 1995a) in comparison to other Balkan countries, which were only studied sporadically (Horvat 1993, 1995b, 1997, Ivković et al. 2012, 2013b, 2014).

Our comparison of Sørensen Similarity indices shows that the FYR Macedonia assemblage has the greatest similarity with the Greek assemblage. This was expected since FYR Macedonia borders with Greece, so they have many species in common. The lowest similarity is with Montenegro, which was not expected as it is geographically much closer to Greece, but this could be due to undersampling of that country (Ivković et al. 2014).

### Concluding remarks

The Greek aquatic Empididae fauna is composed of exclusively Palearctic taxa with the exception of *C. stagnalis*, which is the most widespread clinocerine (known from North America, Asia, and North Africa) (Sinclair 2008). Most of the species are restricted to Europe or South Europe and some of them are only found in the Balkans and Greek islands (e.g., *Dolichocephala zwicki*, Wiedemannia (Chamaedipsia) ariadne, W. (Pseudowiedemannia) microstigma, W. (Wiedemannia) dinarica and *W. artemisa*). Some species have a small area of distribution, occurring in just one or a few sites (e.g., *Chelifera horvati*, *Clinocerella siveci*, *Dolichocephala cretica*, *D. vaillanti*, *Roederiodes malickyi*, Wiedemannia (W.) graeca, *W. iphigeniae*, *W. ljerkae*, *W. nebulosa* and *W. pseudoberthelemyi*), and can be considered as Greek endemics.

There are still some genera of Clinocerinae and Hemerodromiinae that have not been recorded in Greece and that might be present, as they occur in surrounding countries (e.g., *Bergenstammia* Mik, *Chelipoda* Macqaurt and *Phyllodromia* Zetterstedt). Within Greece, most species were reported from the Hellenic Western Balkan Ecoregion; this was expected as this European Ecoregion covers most of the surface area of the country (Illies 1978) and it is considered a biodiversity hotspot (Kryštufek and Reed 2004). The checklist presented here only includes species for which good evidence exists of their presence in Greece. As explained previously, we have omitted any ambiguous or doubtful data and references. This paper may serve as a baseline for planning future work in Greece, but also in surrounding countries for which knowledge of the aquatic dance fly fauna is poor, such as Albania, Bulgaria and Turkey.

## Supplementary Material

XML Treatment for Wiedemannia iphigeniae

XML Treatment for Wiedemannia ljerkae

XML Treatment for Wiedemannia nebulosa

XML Treatment for Wiedemannia pseudoberthelemyi

XML Treatment for Chelifera horvati

## References

[B1] Bezzi M (1904) Empididae novae Palaearcticae ex Museo Nationali Hungarico. Annales Historico-Naturales Musei Nationalis Hungarici 2: 198–202.

[B2] Bezzi M (1905) Clinocerae tres novae ex Europa. Annales Historico-Naturales Musei Nationalis Hungarici 3: 362–366.

[B3] Chvála M (2012) Fauna Europaea: Empididae In: Pape T, Beuk P (Eds) Fauna Europaea: Diptera Brachycera Available from: http://www.fauna-eu.org [Accessed 2 May 2017]

[B4] Clarke KR Gorley RN (2006) PRIMER v6: User Manual/Tutorial. PRIMER-E, Plymouth, 192 pp http://www.primer-e.com/index.htm

[B5] Collin JE (1927) Notes on the Empididae (Diptera) with additions and corrections to the British list. Entomologist’s Monthly Magazine 63: 2–29, 61–67, 93–98.

[B6] Collin JE (1961) Empididae. British flies. Volume 6. University Press, Cambridge, 782 pp.

[B7] Cumming JM Wood DM (2009) Adult morphology and terminology [Chapter] 2. In: Brown BV Borkent A Cumming JM Wood DM Woodley NE Zumbado MA (Eds) Manual of Central American Diptera. Volume 1. NRC Research Press, Ottawa, 9–50.

[B8] Engel EO (1918) Das Dipterengenus *Atalanta* Mg. (*Clinocera* ol.). Deutsche Entomologische Zeitschrift 1918: 1–80, 197–268.

[B9] Engel EO (1939) 28. Empididae. In: Lindner E (Ed.) Die Fliegen der Palaearktischen Region. Band IV (4) [Lieferung 130]. E. Schweizerbart, Stuttgart, 105–152. [Taf. II–VI]

[B10] Engel EO (1940) 28. Empididae. In: Linder, E. (Ed.) Die Fliegen der palaearktischen Region. Band IV (4) [Lieferung 132]. E. Schweizerbart, Stuttgart, 153–192. [Taf. VII–XIII]

[B11] Fallén CF (1816) Empidiae Sveciae. Lundae, 17–34.

[B12] Fallén CF (1826) Supplementum Dipterorum Sveciae. Lundae, 16 pp.

[B13] Haliday AH (1833) Catalogue of Diptera occurring about Holywood in Downshire. The Entomological Magazine 1: 147–180.

[B14] Harkrider JR (2000) Predation of *Neoplasta* Coquillett larvae (Diptera: Empididae) on larval midges in the genus *Rheotanytarsus* Bause (Diptera: Chironomidae). Pan-Pacific Entomologist 76: 176–183.

[B15] Horvat B (1993) Aquatic Empididae fauna (Diptera) in Bosnia and Herzegovina. Scopolia 28: 1–25.

[B16] Horvat B (1995a) Checklist of the aquatic Empididae recorded from Slovenia, with the description of one new species (Diptera). Acta Entomologica Slovenica 3: 25–35.

[B17] Horvat B (1995b) Aquatic Empididae Fauna (Diptera) in Macedonia. Acta Musei Macedonici Scientiarum Naturalium 19: 147–170.

[B18] Horvat B (1997) New records of aquatic Empididae (Diptera) from Macedonia. Studia Dipterologica 4: 491–496.

[B19] Illies J (1978) Limnofauna Europaea. A checklist of the animals inhabiting European inland waters, with account of their distribution and ecology. G. Fischer, Stuttgart, New York and Swets & Zeitlinger, Amsterdam, 532 pp.

[B20] Ivković M Gračan R Horvat B (2013a) Croatian aquatic dance flies (Diptera: Empididae: Clinocerinae and Hemerodromiinae): species diversity, distribution and relationship to surrounding countries. Zootaxa 3686: 255–276. https://doi.org/10.11646/zootaxa.3686.2.7 26473217 10.11646/zootaxa.3686.2.7

[B21] Ivković M Matoničkin Kepčija R Mihaljević Z Horvat B (2007) Assemblage composition and ecological features of aquatic dance flies (Diptera, Empididae) in the Cetina River system, Croatia. Fundamental and Applied Limnology 170: 223–232. https://doi.org/10.1127/1863-9135/2007/0170-0223

[B22] Ivković M Mihaljević Z Miliša M Previšić A (2013b) Aquatic dance flies fauna (Diptera, Empididae: Clinocerinae and Hemerodromiinae) of Montenegro. Natura Croatica 22: 243–252.

[B23] Ivković M Miliša M Mihaljević Z (2010) The aquatic dance flies fauna (Diptera, Empididae: Hemerodromiinae and Clinocerinae) of the Plitvice Lakes National Park. Natura Croatica 19: 133–139.

[B24] Ivković M Plant A (2015) Aquatic insects in the Dinarides: identifying hotspots of endemism and species richness shaped by geological and hydrological history using Empididae (Diptera). Insect Conservation and Diversity 8: 302–312. https://doi.org/10.1111/icad.12113

[B25] Ivković M Plant A Horvat B (2012) A new species of *Wiedemannia* (Diptera: Empididae: Clinocerinae) from Balkan Peninsula. Zootaxa 3478: 581–585.

[B26] Ivković M Vojvoda Zeljko T Previšić A (2014) New records of aquatic Dance flies (Empididae: Clinocerinae and Hemerodromiinae) from Bosnia and Herzegovina. Natura Croatica 23: 401–406.

[B27] Joost W (1981) Beitrag zur Kenntnis der Hemerodromiinae des Kaukasus (1) (Diptera, Empididae). Reichenbachia 19: 183–191.

[B28] Joost W (1982) Beitrag zur Kenntnis der Hemerodromiinae Bulgariens (Insecta, Diptera, Empididae). Faunistische Abhandlungen Staatliches Museum für Tierkunde in Dresden 9: 121–124.

[B29] Kryštufek B Reed JM (2004) Pattern and process in Balkan biodiversity – an overview. In: Griffiths HI Kryštufek B Reed JM (Eds) Balkan Biodiversity: Pattern and Process in the European Hotspot. Kluwer Academic Publishers, Dordrecht, 1–8. https://doi.org/10.1007/978-1-4020-2854-0_1

[B30] Loew H (1869) Beschreibungen europäischer Dipteren. Systematische Beschreibung der bekannten europäischen zweiflügeligen Insecten, von Johann Wilhelm Meigen. Halle 1: 1–310.

[B31] Loew H (1873) Diptera nova, in Pannonia inferiori et in confinibus Daciae regionsibus a Ferd. Kowarzio capta. Berliner Entomologische Zeitschrift 17: 33–52.

[B32] Mandaron P (1964) Un nouveau Diptère récolté en Dauphiné Wiedemannia (Chamaedipsia) aequilobata n. sp. Travaux du Laboratoire d’Hydrobiologie et de Pisiculture de l’Université de Grenoble 56: 81–84.

[B33] McAlpine JF (1981) Morphology and terminology – adults. In: McAlpine JF Peterson BV Shewell GE Teskey HJ Vockeroth JR Wood DM (Coords) Manual of Nearctic Diptera Volume 1. Agriculture Canada Monograph 27: 9–63.

[B34] Meigen JW (1804) Klassifikazion und Beschreibung der europäischen zweiflügeligen Insekten (Diptera Linn.). Erster Band. Reichard, Braunschweig. 152 pp [Abt. I]; 153–314 [Abt. II].

[B35] Mik J (1880) Beschreibung neuer Dipteren. I. Eilf neue europäische *Clinocera*-Arten. Verhandlungen der Kaiserlich-Königlichen Zoologisch-Botanischen Gesellschaft in Wein 30: 347–358.

[B36] Mik J (1889) Eine neue schweizerische Art aus der alten Gattung *Clinocera* Meig. (Ein dipterologischer Beitrag.). Wiener Entomologische Zeitung 8: 71–72. https://doi.org/10.5962/bhl.part.20027

[B37] Oldenberg L (1910) Einige europäische Empididen. Annales Historico-Naturales Musei Nationalis Hungarici 8: 344–352.

[B38] Saigusa T (2006) Homology of wing venation of Diptera. Privately published handout distributed at the 6th International Congress of Dipterology, Fukuoka, Japan, 26 pp.

[B39] Schmidt-Kloiber A Neu PJ Malicky M Pletterbauer F Malicky H Graf W (2017) Aquatic biodiversity in Europe: a unique dataset on the distribution of Trichoptera species with important implications for conservation. Hydrobiologia 797: 11–27. https://doi.org/10.1007/s10750-017-3116-4

[B40] Sinclair BJ (1995) Generic revision of the Clinocerinae (Empididae), and description and phylogenetic relationships of the Trichopezinae, new status (Diptera: Empidoidea). The Canadian Entomologist 127: 665–752. https://doi.org/10.4039/Ent127665-5

[B41] Sinclair BJ (2008) The systematics of New World *Clinocera* Meigen (Diptera: Empididae: Clinocerinae). NRC Research Press, Ottawa, 245 pp https://doi.org/10.1139/9780660198002

[B42] Sinclair BJ Cumming JM (2006) The morphology, higher-level phylogeny and classification of the Empidoidea (Diptera). Zootaxa 1180: 1–178.

[B43] Stuckenberg BR (1999) Antennal evolution in the Brachycera (Diptera), with a reassessment of terminology relating to the flagellum. Studia Dipterologica 6: 33–48.

[B44] Vaillant F (1952) Un empidide destructeur de simulies. Bulletin de la Société Zoologique de France 76 [1951]: 371–379.

[B45] Vaillant F (1953) *Hemerodromia seguyi*, nouvel empidide d’Algérie destructeur de simulies. Hydrobiologia 5: 180–188. http://dx.doi.org/10.1007/bf00023589

[B46] Vaillant F (1957) Contribution à l′étude des Diptères Empididae du Grand-Atlas Marocain. Société des Sciences Naturelles et Physiques du Maroc, Bulletin 36(1)(1956): 61–71.

[B47] Vaillant F (1965) Revision des Empididae Hemerodromiinae de France, d’Espagne et d’Afrique du Nord [Dipt.]. Annales de la Société Entomologique de France 133(1964): 143−173.

[B48] Vaillant F (1967) La répartition des *Wiedemannia* dans les cours d’eau et leur utilisation comme indicateurs de zones écologiques (Diptera, Empididae). Annales de Limnologie 3(2): 267–293. http://dx.doi.org/10.1051/limn/1967016

[B49] Vaillant F (1982) Diptères Empididae Hemerodromiinae nouveaux ou per connus de la région paléarctique (première partie). Bonner Zoologische Beiträge 32 [1981]: 351–408. [published January 1982]

[B50] Vaillant F Vinçon G (1987) Quelques Clinocerini (Diptera, Empididae, Hemerodromiinae) nouveaux ou mal connus des Pyrénées. Annales de Limnologie 22(3)[1986]: 261–275.

[B51] Vaillant F Wagner R (1990) Notes on Empididae (6), *Wiedemannia graeca* sp.n. Aquatic Insects 12: 95–96. http://dx.doi.org/10.1080/01650429009361394

[B52] Wagner R (1981) Über einige Hemerodromiinae vom Balkan und aus der Ägäis. Spixiana 4: 297–304.

[B53] Wagner R (1990) Neue *Wiedemannia*-Arten aus der *rhynchops*-Gruppe (Diptera, Empididae, Clinocerinae). Entomofauna 11: 229−237.

[B54] Wagner R (1995) Empididen aus dem Mittelmeerraum (Diptera, Empididae: Hemerodromiinae und Clinocerinae). Acta Entomologica Slovenica 3: 5−23.

[B55] Wagner R (1997) Diptera Empididae, dance flies. In: Nilsson A (Ed.) Aquatic insects of North Europe, a taxonomic handbook, Volume 2. Apollo Books, Stenstrup, 333–344.

[B56] Wagner R Gathmann O (1996) Long-term studies on aquatic Dance Flies (Diptera, Empididae) 1983–1993: Distribution and size patterns along the stream, abundance changes between years and the influence of environmental factors on the community. Archiv für Hydrobiologie 137: 385–410.

[B57] Wagner R Horvat B (1993) The genus *Roederiodes* Coquillett, 1901 (Diptera, Empididae: Clinocerinae) in Europe, with descriptions of four new species. Bonner Zoologische Beiträge 44: 33–40.

[B58] Walker F (1851) Insecta Britannica, Diptera. Volume 1. Reeve and Benham, London, 314 pp.

[B59] Werner D Pont AC (2003) Dipteran predators of Simuliid blackflies: a worldwide review. Medical and Veterinary Entomology 17: 115–132. http://dx.doi.org/10.1046/j.1365-2915.2003.00431.x 12823828 10.1046/j.1365-2915.2003.00431.x

[B60] Yang D Zhang KY Yao G Zhang JH (2007) World catalog of Empididae (Insecta: Diptera). China Agricultural University Press, Beijing, 599 pp.

[B61] Zetterstedt JW (1838) Sectio tertia. Diptera Dipterologis Scandinaviae amicis et popilaribus carissimus. Insecta Lapponica, Lipsiae (= Leipzig), 477–868.

[B62] Zetterstedt JW (1842) Diptera Scandinaviae deposita et descripta. Lundae (Lund) 1: 1–440.

